# Supplementary material for: Immunogenicity, reactogenicity, and IgE-mediated immune responses of a mixed whole-cell and acellular pertussis vaccine schedule in Australian infants: A randomised, double-blind, noninferiority trial
Source: PLoS Med. 2024 Jun 10;21(6):e1004414. doi: 10.1371/journal.pmed.1004414 (PMC11198910; doi:10.1371/journal.pmed.1004414)
Supplement: S1 Text — (PDF) [file pmed.1004414.s001.pdf]

Supplemental Information

Immunogenicity, reactogenicity, and IgE-mediated immune responses of a mixed whole-cell and acellular pertussis vaccine schedule in Australian infants: a randomised, double-blind, non-inferiority trial

Table of contents

A Statistical Analysis Methods **4**
A.1 Concentrations 4
A.2 Seropositivity, sensitisation, and quantitation 4
A.3 4-fold-rise 5

B Enrolments **6**

C IgG Concentrations **7**
C.1 Missingness 7
C.2 Sample Summaries 7
C.3 Analyses of IgG Concentrations 9
C.4 Analyses of IgG Sero-positivity 10
C.5 Analyses of IgG 4-fold-rise 11
C.6 Sample Summaries (per-protocol) 12
C.7 Analyses of IgG Concentrations (per-protocol) 13
C.8 Analyses of IgG Sero-positivity (per-protocol) 14
C.9 Analyses of IgG 4-fold-rise (per-protocol) 15

D IgE Concentrations **16**
D.1 Missingness 16
D.2 Sample Summaries 18
D.3 Analyses of IgE Concentrations 19
D.4 Analyses of IgE Sensitisation 20
D.5 Analyses of IgE Quantitation 21
D.6 Sample Summaries (per-protocol) 22
D.7 Analyses of IgE Concentrations (per-protocol) 23
D.8 Analyses of IgE Sensitisation (per-protocol) 24
D.9 Analyses of IgE Quantitation (per-protocol) 25

E Reactogenicity **26**
E.1 Solicited Systemic Reactions 26
E.2 Solicited Injection Site Reactions 34
E.3 Parental Satisfaction 39

References **40**

List of Figures

A Cumulative (line) and monthly (bar) enrolments for the sentinel cohort. 6
B IgG scatterplots by type and assigned vaccination schedule, pertussis and other antigens. Diagonal dotted line indicates equality. 8
C Marginal posterior density for geometric mean ratio (wP vs aP) by vaccination age, pertussis antigens. 9
D Posterior density of probability and difference for IgG seropositivity by antigen, age, and pertussis schedule for pertussis and other antigens. Excludes antigens with 100% seropositivity. 10
E Posterior standardised probability and difference for 4-fold rise in IgG from 6-months to 7-months of age by antigen. 11
F Marginal posterior density for geometric mean ratio (wP vs aP) by vaccination age, pertussis antigens (per-protocol). 13
G Posterior density for standardised probability and difference of IgG seropositivity by antigen, age, and pertussis schedule for pertussis and other antigens (per-protocol). 14
H Posterior standardised probability and difference for 4-fold rise in IgG from 6-months to 7-months of age by antigen (per-protocol). 15
I IgE boxplots by allergen type, age, and assigned vaccination schedule. Horizontal dotted line indicates 4-fold rise. 18
J Posterior density for geometric mean ratio by vaccination age for total IgE. 19
K Posterior density of standardised probability and difference for IgE sensitisation by age and assigned treatment group. 20
L Posterior density of standardised probability and difference for IgE quantitation by age and assigned treatment group. 21
M Posterior density for geometric mean ratio by vaccination age for total IgE (per-protocol). 23
N Posterior density of standardised probability and difference for IgE sensitisation by age and assigned treatment group (per-protocol). 24
O Posterior density standardised probability and difference for IgE quantitation by age and assigned treatment group (per-protocol). 25
P Irritability daily intensity grades, day 0 to 6 by vaccination age and assigned treatment. 27
Q Vomiting daily intensity grades, day 0 to 6 by vaccination age and assigned treatment. 28
R Diarrhoea daily intensity grades, day 0 to 6 by vaccination age and assigned treatment. 29
S Decreased feeding daily intensity grades, day 0 to 6 by vaccination age and assigned treatment. 30
T Drowsiness daily intensity grades, day 0 to 6 by vaccination age and assigned treatment. 31
U Restlessness daily intensity grades, day 0 to 6 by vaccination age and assigned treatment. 32
V Fever day 0 to 6 by vaccination age and assigned treatment. 33
W Injection site erythema by vaccination age, vaccine, and assigned treatment. 35
X Injection site swelling by vaccination age, vaccine, and assigned treatment. 36
Y Injection site induration by vaccination age, vaccine, and assigned treatment. 37
Z Distribution of responses to "I would be willing to have another child have this combination of immunisations at this age" by vaccination occassion and assigned treatment. 39

List of Tables

A Missingness pattern for IgG by assigned treatment group. "X" marks observed values and "-" missing values. Missingness was the same for all antigen types. 7
B Sample summaries of IgG concentration by antigen type, age, and assigned treatment group. 7

C Posterior summaries of sero-positivity models for pertussis antigens. Excludes antigens with 100% sample seropositivity. . . . 10
D Posterior standardised probability, difference, and conditional odds ratio for 4-fold rise in IgG from 6-months to 7-months of age by antigen. . . . 11
E Sample IgG concentration summaries by antigen type, age, and assigned treatment (per-protocol). . . . 12
F IgG GMR posterior summaries and non-inferiority probability, pertussis antigens (per-protocol). . . . 13
G Summary of sero-positivity model for pertussis antigens (per-protocol). . . . 14
H Posterior standardised probability, difference, and conditional odds ratio for 4-fold rise in IgG from 6-months to 7-months of age by antigen (per-protocol). . . . 15
I Missingness pattern for total IgE by assigned schedule. "X" marks observed values and "-" missing values. . . . 16
J Missingness pattern for tetanus-toxoid IgE by assigned schedule. "X" marks observed values and "-" missing values. . . . 16
K Missingness pattern for egg white IgE by assigned schedule. "X" marks observed values and "-" missing values. . . . 16
L Missingness pattern for whole egg IgE by assigned schedule. "X" marks observed values and "-" missing values. . . . 16
M Reasons for missing total IgE concentration data by occassion and treatment group. 17
N Sample IgE concentration summaries (kU/L) by allergen type and assigned vaccination schedule. . . . 18
O IgE concentration posterior summaries for Geometric mean ratio (GMR) and geometric mean fold-rise ratio (GMFR) (wP vs aP). . . . 19
P Summary of sensitisation model for tetanus toxoid IgE. . . . 20
Q Sample IgE concentration summaries (kU/L) by allergen type and assigned vaccination schedule for per-protocol outcomes. . . . 22
R IgE concentration posterior summaries for Geometric mean ratio (GMR) and geometric mean fold-rise ratio (GMFR), wP vs aP (per-protocol). . . . 23
S Summary of sensitisation model for tetanus toxoid IgE (per-protocol). . . . 24
T Posterior summaries of quantitation model for IgE concentrations (per-protocol). . . . 25
U Highest intensity grade for systemic reactions following each vaccination (day 0 to 6) by assigned treatment. . . . 26
V Experience of any severe intensity systemic reaction following vaccination (day 0 to 6) by assigned treatment. . . . 26
W Highest fever following each vaccination by assigned treatment. . . . 33
X Maximum injection site reaction size in the 7 days following vaccination, by vaccination age, vaccine, and assigned treatment. . . . 34
Y Maximum injection site pain in the 7 days following vaccination, by vaccination age, vaccine, and assigned treatment. . . . 38
Z Number and percentage of respondents by response to the statement: "I would be willing to have another child have this combination of immunisations at this age." . 39

A Statistical Analysis Methods

The analysis methods were as outlined in the SAP (Totterdell et al. 2022). Model details are provided here for reference. In addition to the term for treatment group, all models reported in the main manuscript and this supplement were adjusted for the covariates: sex (male or female), breast feeding status (complete, partial, not at all), first born child (yes or no), mode of delivery (cesarean or not), family history of atopy (yes or no), and parental income ($\leq$ \$87,000, \$87,001 – \$180,000, $>$ \$180,000). Results from unadjusted models were generally consistent with the adjusted models, therefore, results from unadjusted models are not reported.

A.1 Concentrations

The IgG and IgE concentrations for each antigen and allergen were analysed independently assuming that the log-transformed values follow a multivariate normal model with unstructured covariance matrix shared across all individuals. Specifically, for participants, $i = 1, \dots, n$, measured on occasions, $j = 1, \dots, J$, with covariates, x_{ip}, for $p = 1, \dots, P$ and concentration to a given antigen/allergen Y_i, we assumed the model

$$
\begin{aligned}
\log_{10}(Y_i) &\sim \text{Normal}(\mu_i, \Sigma) \\
\mu_{ij} &= \beta_{j0} + \sum_{p=1}^{P} x_{ip}\beta_{jp} \\
\Sigma &= \text{diag}(\sigma)\Omega\text{diag}(\sigma) \\
\beta_{jp} &\sim \text{Normal}(0, 10^2), \quad j = 1, \dots, J, \; p = 1, \dots, P \\
\Omega &\sim \text{LKJ}(1) \\
\sigma_j &\sim \text{Cauchy}^+(0, 10), \quad j = 1, \dots, J.
\end{aligned}
$$

In the analysis of IgE concentrations some values were reported as 0.00 kU/L. These values were treated as left-censored in the model at 0.005 kU/L to satisfy $Y_i > 0$.

A.2 Seropositivity, sensitisation, and quantitation

The binary outcomes related to IgG seropositivity, IgE sensitisation, and IgE quantitation were analysed by independent logistic regression models with random intercepts for individuals. Denoting by Y_{ij} the binary outcome for participant $i = 1, \dots, n$, measured on occasions, $j = 1, \dots, J$, with model covariates, x_{ip}, for $p = 1, \dots, P$, the assumed model was

$$
\begin{aligned}
Y_{ij} &\sim \text{Bernoulli}(p_{ij}) \\
p_{ij} &= \text{logit}^{-1}(\eta_{ij}) \\
\eta_{ij} &= (\beta_{j0} + \gamma_i) + \sum_{p=1}^{P} x_{ip}\beta_{jp} \\
\gamma_i|\tau &\sim \text{Normal}(0, \tau^2), \quad i = 1, \dots, n \\
\beta_{jp} &\sim \text{Student-}t(4, 0, 1.75^2), \quad j = 1, \dots, J, \ p = 1, \dots, P \\
\tau &\sim \text{Exponential}(1)
\end{aligned}
$$

As a measure of effect we reported the conditional odds ratio (cOR), the marginal odds ratio (mOR), and the event probability (and the difference) standardised to the covariate distribution observed in the trial sample. The marginal odds ratios were calculated by integrating out the random intercepts as outlined in Hedeker et al. (2018) using the R package `brmsmargins` (Wiley and Hedeker 2022). Letting, x_{i1} denote the treatment, and $x_{i,2:p}$ the other model covariates, the standardised difference for occasion j was calculated as

$$
\begin{aligned}
p_j(x_i) &= \mathbb{P}(Y_j = 1 | X_i = x_i) \\
&= \int_{-\infty}^{\infty} \text{logit}^{-1}\left(\beta_{j0} + \gamma + \sum_{p=1}^{P} x_{ip}\beta_{jp}\right) f(\gamma)\, \mathrm{d}\gamma \\
D_j &= n^{-1} \sum_{i=1}^{n} p_j(1, x_{i,2:p}) - p_j(0, x_{i,2:p})
\end{aligned}
$$

again via `brmsmargins`. The baseline covariates of subject's with missing outcomes were used in calculating the standardised event probability.

A.3 4-fold-rise

The binary outcomes for the event of a 4-fold rise in concentration from 6-months to 7-months for each antigen were analysed by assuming independent logistic regression models. Denoting by Y_i the binary outcome for participant $i = 1, \dots, n$, with model covariates, x_{ip}, for $p = 1, \dots, P$, the assumed model was

$$
\begin{aligned}
Y_i &\sim \text{Bernoulli}(p_i) \\
p_i &= \text{logit}^{-1}(\eta_i) \\
\eta_i &= \beta_0 + \sum_{p=1}^{P} x_{ip}\beta_p \\
\beta_p &\sim \text{Student-}t(4, 0, 1.75^2), \quad j = 1, \dots, J, \ p = 1, \dots, P
\end{aligned}
$$

As a measure of effect we reported the conditional odds ratio and the event probability (and difference) standardised to the covariate distribution observed in the trial sample.

B Enrolments

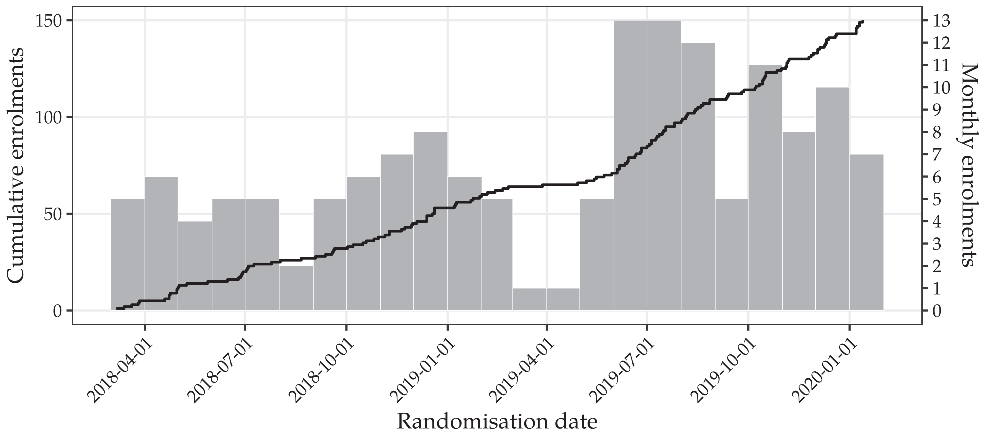

Figure A: Cumulative (line) and monthly (bar) enrolments for the sentinel cohort.

C IgG Concentrations

C.1 Missingness

Table A: Missingness pattern for IgG by assigned treatment group. "X" marks observed values and "-" missing values. Missingness was the same for all antigen types.

		Count (%)	
6-month	7-month	aP	wP
X	X	66 (44%)	65 (43%)
X	-	6 (4%)	3 (2%)
-	X	3 (2%)	2 (1%)
-	-	0 (0%)	5 (3%)

C.2 Sample Summaries

Table B: Sample summaries of IgG concentration by antigen type, age, and assigned treatment group.

	aP (n = 75)						wP (n = 75)					
	n	GMC[1]	Q1–Q3[2]	Min–Max	S+[3]	4-fold[4]	n	GMC[1]	Q1–Q3[2]	Min–Max	S+[3]	4-fold[4]
PT												
6-month	72	13.66	7.22–22.5	2.31–75.1	90%	—	68	4.94	3.42–6.90	1.83–18.3	49%	—
7-month	69	27.69	17.5–45.8	5.14–145	100%	11%	67	26.38	15.2–39.9	3.59–152	99%	74%
PRN												
6-month	72	15.21	7.28–32.2	2.15–144	86%	—	68	14.87	7.65–28.8	2.33–190	93%	—
7-month	69	51.19	30.6–81.7	7.77–378	100%	42%	67	38.36	20.3–68.2	7.58–610	100%	29%
FIM2/3												
6-month	72	5.40	1.21–20.2	0.02–284	51%	—	68	9.77	4.49–24.9	0.24–133	72%	—
7-month	69	3.89	0.86–12.3	0.09–177	43%	2%	67	7.12	3.06–19.5	0.12–79.9	58%	0%
FHA												
6-month	72	45.24	27.8–77.8	8.92–193	100%	—	68	15.02	8.86–24.6	4.47–180	99%	—
7-month	69	82.39	52.9–123	19.8–563	100%	8%	67	56.12	33.9–99.9	9.68–240	100%	54%
DT												
6-month	72	0.14	0.08–0.23	0.03–1.01	62%	—	68	0.15	0.09–0.23	0.03–2.16	71%	—
7-month	69	0.61	0.42–0.98	0.08–3.20	99%	56%	67	0.86	0.51–1.29	0.24–4.84	100%	72%
TT												
6-month	72	0.70	0.44–1.19	0.16–3.60	100%	—	68	0.78	0.49–1.17	0.19–2.84	100%	—
7-month	69	1.41	0.95–2.02	0.44–6.86	100%	9%	67	1.63	1.14–2.47	0.38–9.84	100%	12%

[1]Geometric mean concentration
[2]Q1 - 25th sample percentile, Q3 - 75th sample percentile
[3]Seropositive at specified level
[4]4-fold rise from 6-month to 7-month

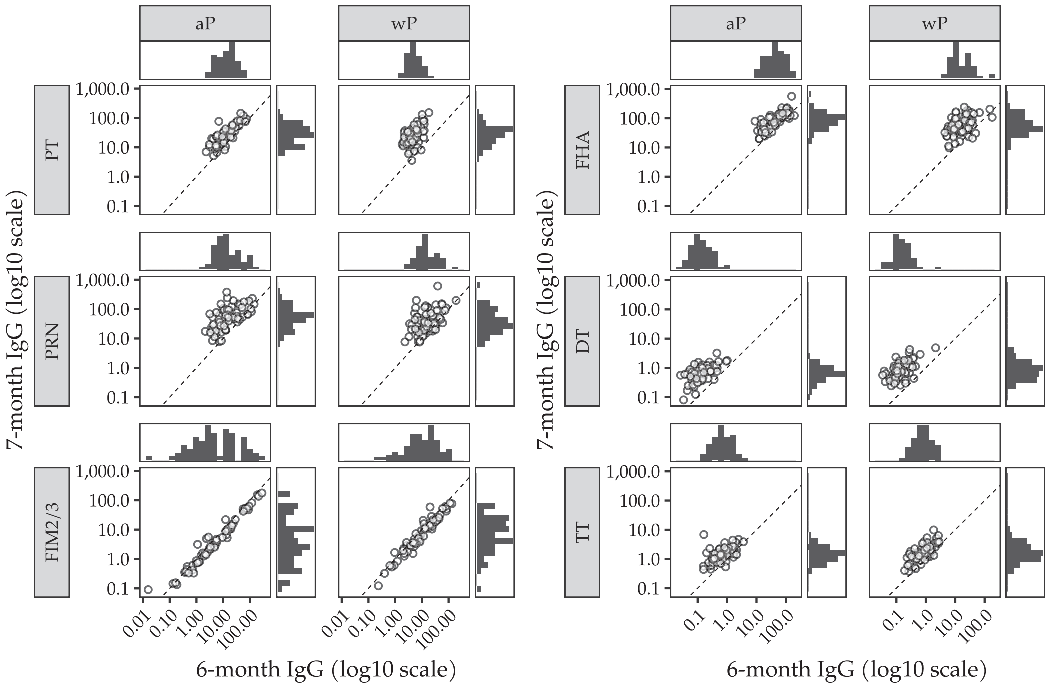

Figure B: IgG scatterplots by type and assigned vaccination schedule, pertussis and other antigens. Diagonal dotted line indicates equality.

C.3 Analyses of IgG Concentrations

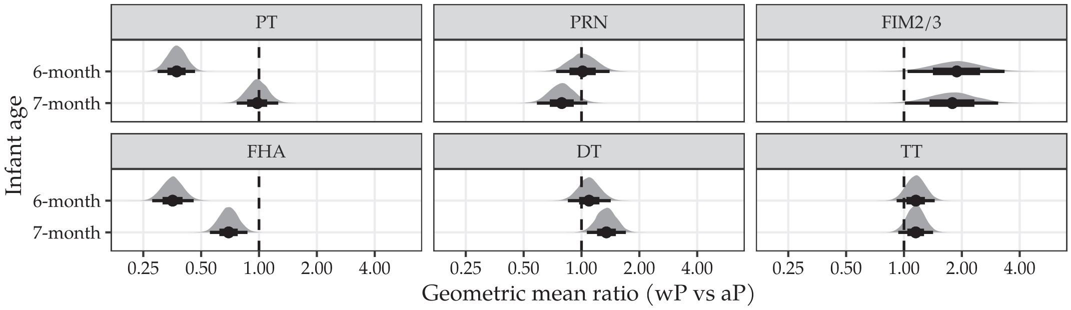

Figure C: Marginal posterior density for geometric mean ratio (wP vs aP) by vaccination age, pertussis antigens.

C.4 Analyses of IgG Sero-positivity

Table C: Posterior summaries of sero-positivity models for pertussis antigens. Excludes antigens with 100% sample seropositivity.

	Odds ratio, Median (95% CrI)		Standardised Pr(S+), Median (95% CrI)			
	cOR	mOR	aP	wP	wP − aP	Pr(wP − aP > 0)
**PT**						
6-month	0.05 (0.00, 0.17)	0.09 (0.03, 0.23)	0.89 (0.81, 0.95)	0.50 (0.38, 0.62)	−0.39 (−0.52, −0.26)	0.00
7-month	0.18 (0.01, 4.26)	0.20 (0.01, 3.91)	1.00 (0.96, 1.00)	0.98 (0.93, 1.00)	−0.01 (−0.06, 0.02)	0.13
**PRN**						
6-month	2.17 (0.57, 10.4)	1.90 (0.64, 6.41)	0.87 (0.78, 0.93)	0.92 (0.84, 0.97)	0.05 (−0.04, 0.15)	0.87
7-month	3.51 (0.13, 267)	3.38 (0.14, 203)	0.99 (0.96, 1.00)	1.00 (0.97, 1.00)	0.00 (−0.01, 0.04)	0.78
**FIM2/3**						
6-month	14.2 (1.06, 491)	1.73 (1.01, 3.50)	0.54 (0.41, 0.66)	0.67 (0.54, 0.78)	0.12 (0.00, 0.28)	0.98
7-month	6.06 (0.30, 180)	1.44 (0.80, 2.84)	0.46 (0.33, 0.59)	0.55 (0.42, 0.68)	0.08 (−0.05, 0.24)	0.87
**DT**						
6-month	1.84 (0.51, 8.60)	1.41 (0.71, 2.90)	0.63 (0.50, 0.74)	0.70 (0.58, 0.81)	0.07 (−0.07, 0.21)	0.84
7-month	5.04 (0.22, 352)	3.89 (0.30, 208)	0.98 (0.93, 1.00)	0.99 (0.96, 1.00)	0.01 (−0.02, 0.06)	0.84

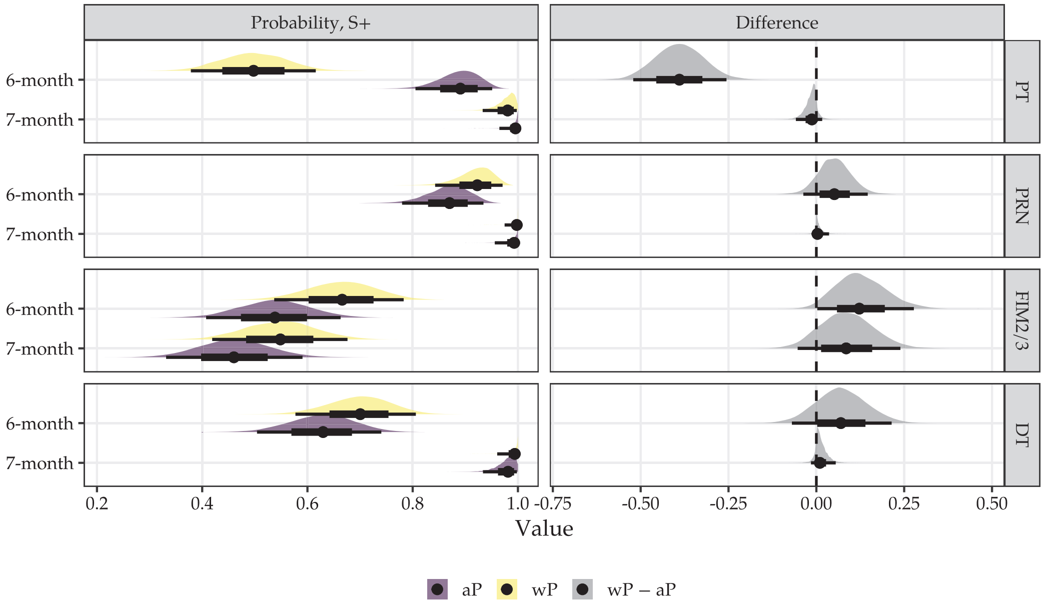

Figure D: Posterior density of probability and difference for IgG seropositivity by antigen, age, and pertussis schedule for pertussis and other antigens. Excludes antigens with 100% seropositivity.

C.5 Analyses of IgG 4-fold-rise

Table D: Posterior standardised probability, difference, and conditional odds ratio for 4-fold rise in IgG from 6-months to 7-months of age by antigen.

	Standardised Pr(4-fold rise), Median (95% CrI)				
	aP	wP	wP − aP	Pr(wP − aP > 0)	OR, wP/aP
PT	0.11 (0.05, 0.20)	0.73 (0.62, 0.82)	0.61 (0.48, 0.73)	1.00	29.0 (11.2, 86.9)
PRN	0.42 (0.31, 0.53)	0.30 (0.21, 0.41)	−0.11 (−0.26, 0.04)	0.07	0.57 (0.26, 1.21)
FIM2/3	0.01 (0.00, 0.05)	0.01 (0.00, 0.04)	0.00 (−0.04, 0.02)	0.27	0.46 (0.01, 5.50)
FHA	0.08 (0.04, 0.16)	0.53 (0.42, 0.65)	0.45 (0.31, 0.58)	1.00	15.3 (5.83, 47.0)
DT	0.57 (0.45, 0.68)	0.71 (0.60, 0.81)	0.15 (−0.01, 0.30)	0.97	1.99 (0.96, 4.15)
TT	0.10 (0.04, 0.18)	0.12 (0.06, 0.20)	0.02 (−0.07, 0.12)	0.69	1.31 (0.44, 4.17)

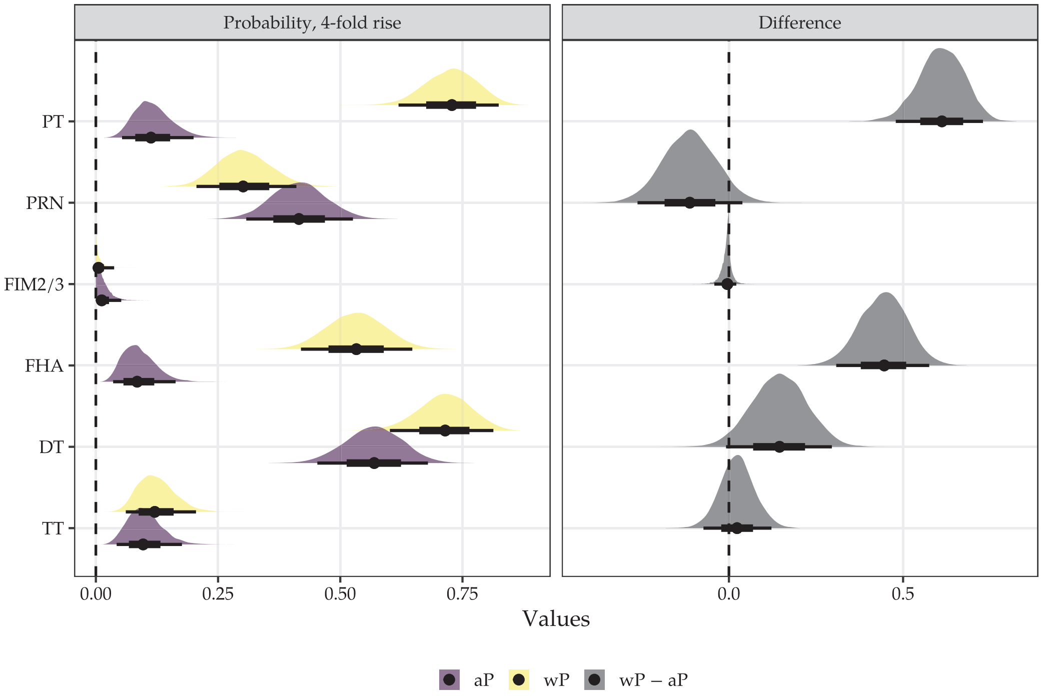

Figure E: Posterior standardised probability and difference for 4-fold rise in IgG from 6-months to 7-months of age by antigen.

C.6 Sample Summaries (per-protocol)

Table E: Sample IgG concentration summaries by antigen type, age, and assigned treatment (per-protocol).

	aP						wP					
	n	GMC[1]	Q1–Q3[2]	Min–Max	S+[3]	4-fold[4]	n	GMC[1]	Q1–Q3[2]	Min–Max	S+[3]	4-fold[4]
PT												
6-month	67	13.63	7.11–22.5	2.31–75.1	91%	—	65	4.94	3.43–6.89	1.83–18.3	48%	—
7-month	58	28.10	18.8–45.0	6.65–145	100%	12%	61	26.53	15.3–39.7	3.59–152	98%	75%
PRN												
6-month	67	15.00	7.26–30.6	2.15–144	87%	—	65	15.25	7.76–30.1	2.33–190	94%	—
7-month	58	53.38	32.2–94.9	7.77–378	100%	48%	61	37.55	20.2–55.5	7.58–610	100%	29%
FIM2/3												
6-month	67	6.22	1.41–21.7	0.02–284	55%	—	65	9.43	4.00–24.5	0.24–133	72%	—
7-month	58	4.03	0.97–11.4	0.09–177	43%	2%	61	7.01	2.83–20.6	0.12–76.1	59%	0%
FHA												
6-month	67	46.11	30.0–76.5	8.92–193	100%	—	65	14.65	8.92–23.6	4.47–180	98%	—
7-month	58	85.32	57.0–124	19.8–563	100%	9%	61	53.03	33.5–96.9	9.68–240	100%	54%
DT												
6-month	67	0.13	0.08–0.22	0.03–1.01	60%	—	65	0.14	0.09–0.21	0.03–2.16	69%	—
7-month	58	0.62	0.39–0.97	0.08–3.20	98%	59%	61	0.84	0.52–1.18	0.24–4.24	100%	75%
TT												
6-month	67	0.67	0.41–1.07	0.16–3.60	100%	—	65	0.79	0.50–1.19	0.19–2.84	100%	—
7-month	58	1.36	0.92–1.98	0.44–6.86	100%	11%	61	1.63	1.17–2.38	0.38–9.84	100%	14%

[1] Geometric mean concentration
[2] Q1 - 25th sample percentile, Q3 - 75th sample percentile
[3] Seropositive at specified level
[4] 4-fold rise from 6-month to 7-month

C.7 Analyses of IgG Concentrations (per-protocol)

Table F: IgG GMR posterior summaries and non-inferiority probability, pertussis antigens (per-protocol).

	Mean ± std	Median	95% CrI	Pr(> 1)	Pr(> 2/3)
PT					
6-month	0.37 ± 0.04	0.37	(0.29, 0.46)	0.00	0.00
7-month	0.95 ± 0.13	0.94	(0.73, 1.21)	0.33	1.00
7/6-month (GMFR)	2.58 ± 0.28	2.56	(2.07, 3.17)	1.00	—
PRN					
6-month	1.08 ± 0.18	1.06	(0.77, 1.47)	0.64	1.00
7-month	0.76 ± 0.12	0.75	(0.54, 1.03)	0.04	0.79
7/6-month (GMFR)	0.72 ± 0.12	0.71	(0.51, 0.98)	0.02	—
FIM2/3					
6-month	1.60 ± 0.50	1.52	(0.85, 2.78)	0.92	1.00
7-month	1.56 ± 0.46	1.50	(0.85, 2.65)	0.92	1.00
7/6-month (GMFR)	0.98 ± 0.07	0.98	(0.85, 1.13)	0.40	—
FHA					
6-month	0.34 ± 0.04	0.33	(0.26, 0.43)	0.00	0.00
7-month	0.64 ± 0.08	0.64	(0.50, 0.81)	0.00	0.40
7/6-month (GMFR)	1.93 ± 0.25	1.92	(1.50, 2.46)	1.00	—
DT					
6-month	1.13 ± 0.15	1.12	(0.87, 1.44)	0.80	1.00
7-month	1.34 ± 0.17	1.33	(1.03, 1.70)	0.99	1.00
7/6-month (GMFR)	1.20 ± 0.15	1.19	(0.94, 1.51)	0.93	—
TT					
6-month	1.21 ± 0.15	1.20	(0.94, 1.52)	0.93	1.00
7-month	1.17 ± 0.14	1.17	(0.93, 1.46)	0.91	1.00
7/6-month (GMFR)	0.98 ± 0.11	0.97	(0.79, 1.20)	0.41	—

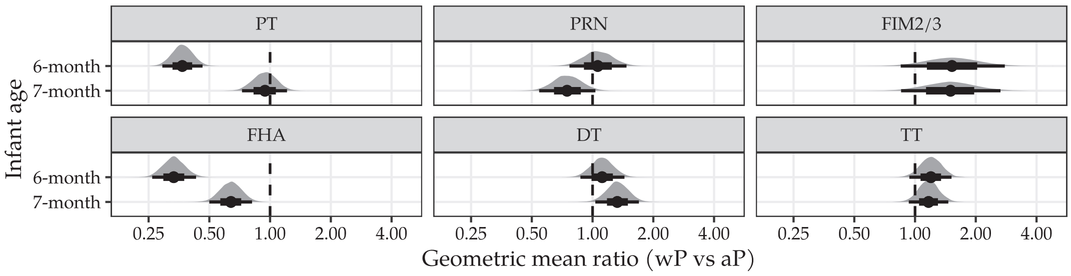

Figure F: Marginal posterior density for geometric mean ratio (wP vs aP) by vaccination age, pertussis antigens (per-protocol).

C.8 Analyses of IgG Sero-positivity (per-protocol)

Table G: Summary of sero-positivity model for pertussis antigens (per-protocol).

	Odds ratio, Median (95% CrI)		Standardised Pr(S+), Median (95% CrI)			
	cOR	mOR	aP	wP	wP − aP	Pr(wP − aP > 0)
PT						
6-month	0.04 (0.00, 0.16)	0.08 (0.03, 0.22)	0.90 (0.81, 0.96)	0.49 (0.36, 0.61)	−0.41 (−0.55, −0.27)	0.00
7-month	0.18 (0.00, 4.42)	0.19 (0.01, 4.13)	0.99 (0.96, 1.00)	0.98 (0.92, 1.00)	−0.01 (−0.07, 0.02)	0.14
PRN						
6-month	2.65 (0.66, 13.6)	2.32 (0.72, 8.65)	0.87 (0.78, 0.94)	0.94 (0.86, 0.98)	0.06 (−0.03, 0.16)	0.91
7-month	4.08 (0.15, 257)	3.86 (0.15, 256)	0.99 (0.95, 1.00)	1.00 (0.97, 1.00)	0.00 (−0.01, 0.04)	0.81
FIM2/3						
6-month	7.36 (0.57, 171)	1.52 (0.90, 3.01)	0.57 (0.44, 0.70)	0.67 (0.54, 0.79)	0.09 (−0.02, 0.24)	0.94
7-month	4.77 (0.21, 129)	1.39 (0.74, 2.85)	0.47 (0.34, 0.60)	0.54 (0.41, 0.67)	0.07 (−0.07, 0.23)	0.85
DT						
6-month	2.13 (0.63, 9.53)	1.55 (0.79, 3.22)	0.59 (0.47, 0.71)	0.69 (0.56, 0.79)	0.09 (−0.05, 0.24)	0.90
7-month	5.52 (0.28, 328)	4.26 (0.36, 200)	0.98 (0.92, 1.00)	0.99 (0.95, 1.00)	0.01 (−0.01, 0.07)	0.87

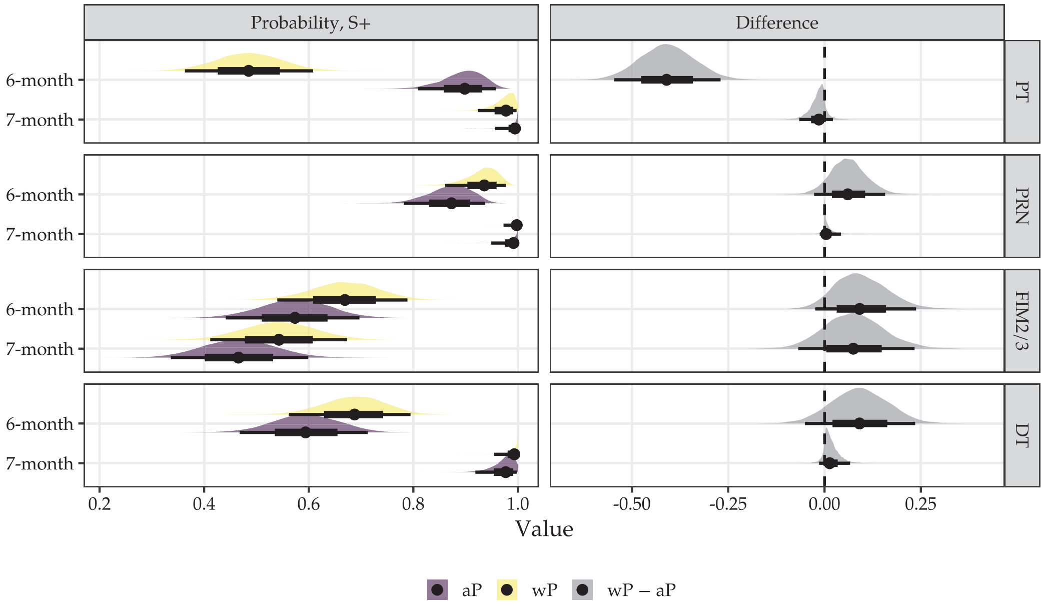

Figure G: Posterior density for standardised probability and difference of IgG seropositivity by antigen, age, and pertussis schedule for pertussis and other antigens (per-protocol).

C.9 Analyses of IgG 4-fold-rise (per-protocol)

Table H: Posterior standardised probability, difference, and conditional odds ratio for 4-fold rise in IgG from 6-months to 7-months of age by antigen (per-protocol).

	Standardised Pr(4-fold rise), Median (95% CrI)				
	aP	wP	wP − aP	Pr(wP − aP > 0)	OR, wP/aP
PT	0.13 (0.06, 0.23)	0.74 (0.62, 0.83)	0.60 (0.45, 0.73)	1.00	24.4 (9.20, 75.7)
PRN	0.47 (0.35, 0.59)	0.30 (0.20, 0.42)	−0.16 (−0.32, 0.00)	0.02	0.44 (0.20, 0.99)
FIM2/3	0.01 (0.00, 0.06)	0.01 (0.00, 0.04)	−0.01 (−0.05, 0.02)	0.26	0.44 (0.01, 5.61)
FHA	0.10 (0.04, 0.19)	0.54 (0.41, 0.65)	0.43 (0.28, 0.57)	1.00	12.6 (4.67, 40.9)
DT	0.60 (0.47, 0.71)	0.74 (0.63, 0.84)	0.14 (−0.02, 0.30)	0.96	2.03 (0.91, 4.61)
TT	0.11 (0.05, 0.20)	0.14 (0.07, 0.23)	0.02 (−0.08, 0.13)	0.66	1.28 (0.43, 4.05)

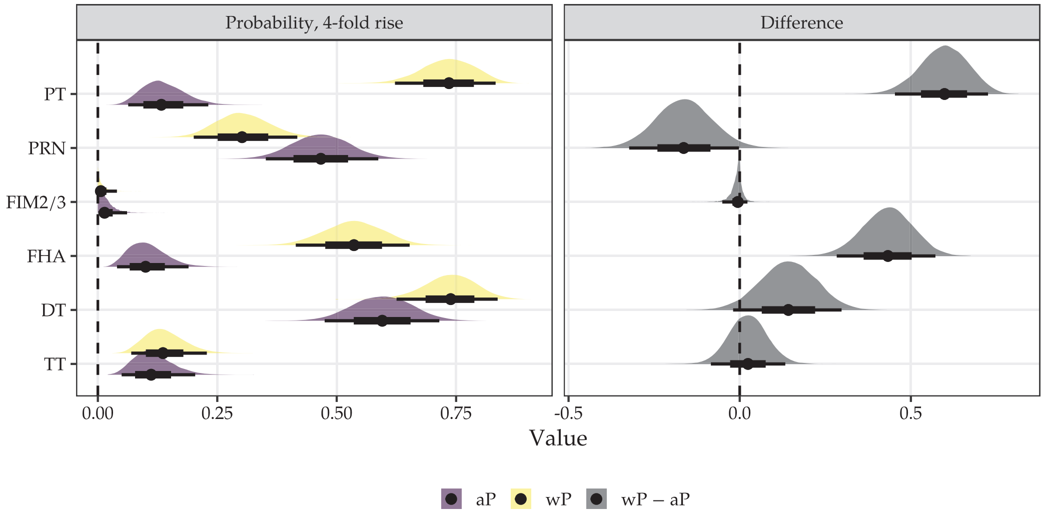

Figure H: Posterior standardised probability and difference for 4-fold rise in IgG from 6-months to 7-months of age by antigen (per-protocol).

D IgE Concentrations

D.1 Missingness

Missingness varied to a small degree by the allergen being assessed as shown in Table I, Table J, Table K, and Table L.

Table I: Missingness pattern for total IgE by assigned schedule. "X" marks observed values and "-" missing values.

		Count (%)	
6-month	7-month	aP	wP
X	X	60 (40%)	52 (35%)
X	-	7 (5%)	9 (6%)
-	X	6 (4%)	5 (3%)
-	-	2 (1%)	9 (6%)

Table J: Missingness pattern for tetanus-toxoid IgE by assigned schedule. "X" marks observed values and "-" missing values.

		Count (%)	
6-month	7-month	aP	wP
X	X	60 (40%)	52 (35%)
X	-	7 (5%)	9 (6%)
-	X	6 (4%)	5 (3%)
-	-	2 (1%)	9 (6%)

Table K: Missingness pattern for egg white IgE by assigned schedule. "X" marks observed values and "-" missing values.

		Count (%)	
6-month	7-month	aP	wP
X	X	60 (40%)	52 (35%)
X	-	7 (5%)	9 (6%)
-	X	7 (5%)	5 (3%)
-	-	1 (1%)	9 (6%)

Table L: Missingness pattern for whole egg IgE by assigned schedule. "X" marks observed values and "-" missing values.

		Count (%)	
6-month	7-month	aP	wP
X	X	60 (40%)	51 (34%)
X	-	7 (5%)	9 (6%)
-	X	7 (5%)	6 (4%)
-	-	1 (1%)	9 (6%)

Table M: Reasons for missing total IgE concentration data by occassion and treatment group.

	6-month			7-month		
Reason	aP	wP	Total	aP	wP	Total
Insufficient sample	6	7	13	2	9	11
Venepuncture unsuccessful	2	5	7	4	3	7
Did not attend	0	1	1	3	3	6
Withdrawn from study	0	1	1	0	1	1
Missing	0	0	0	0	2	2
Total	8	14	22	9	18	27

D.2 Sample Summaries

Table N: Sample IgE concentration summaries (kU/L) by allergen type and assigned vaccination schedule.

	aP						wP					
	n	GMC[1]	Q1–Q3	Min–Max	≥0.01	≥0.1	n	GMC[1]	Q1–Q3	Min–Max	≥0.01	≥0.1
Total IgE												
6-month	67	4.76	2.34–10.11	0.40–121.43	100%	100%	61	6.11	2.40–12.43	0.19–141.38	100%	100%
7-month	66	6.36	2.69–11.72	0.52–241.99	100%	100%	57	7.71	2.66–16.30	0.52–148.70	100%	100%
Tetanus-toxoid												
6-month	67	0.00	0.00–0.00	0.00–0.22	12%	1%	61	0.00	0.00–0.00	0.00–1.71	21%	7%
7-month	66	0.00	0.00–0.00	0.00–0.20	21%	3%	57	0.00	0.00–0.00	0.00–0.82	21%	7%
Egg white												
6-month	67	0.00	0.00–0.02	0.00–14.91	36%	18%	61	0.00	0.00–0.02	0.00–3.00	26%	20%
7-month	67	0.00	0.00–0.04	0.00–39.50	54%	24%	57	0.00	0.00–0.03	0.00–4.51	42%	19%
Whole egg												
6-month	67	0.00	0.00–0.02	0.00–14.85	40%	18%	60	0.00	0.00–0.04	0.00–3.51	42%	22%
7-month	67	0.00	0.00–0.04	0.00–33.70	52%	22%	57	0.00	0.00–0.04	0.00–5.16	40%	21%

[1]Geometric mean concentration. Values of 0.00 set to 0.005 for calculation.

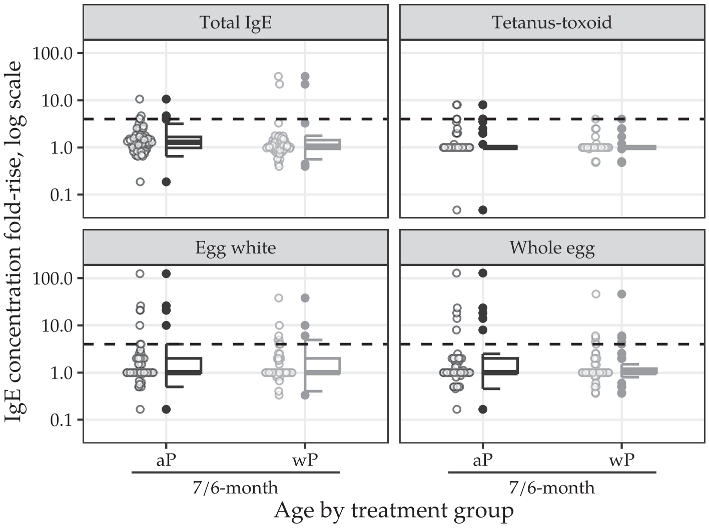

Figure I: IgE boxplots by allergen type, age, and assigned vaccination schedule. Horizontal dotted line indicates 4-fold rise.

D.3 Analyses of IgE Concentrations

Table O: IgE concentration posterior summaries for Geometric mean ratio (GMR) and geometric mean fold-rise ratio (GMFR) (wP vs aP).

	Mean $\pm$ std	Median	95% CrI	Pr(<1)
Total IgE				
6-month	1.29 $\pm$ 0.28	1.26	(0.82, 1.91)	0.14
7-month	1.17 $\pm$ 0.25	1.14	(0.75, 1.72)	0.27
7/6-month (GMFR)	0.92 $\pm$ 0.12	0.91	(0.70, 1.18)	0.77
Tetanus toxoid				
6-month	28.13 $\pm$ 460.03	4.44	(0.27, 125.05)	0.14
7-month	2.53 $\pm$ 9.35	1.08	(0.09, 12.40)	0.47
7/6-month (GMFR)	0.35 $\pm$ 0.42	0.24	(0.03, 1.32)	0.95
Egg white				
6-month	0.67 $\pm$ 0.89	0.40	(0.05, 2.97)	0.82
7-month	0.90 $\pm$ 0.78	0.68	(0.15, 2.91)	0.70
7/6-month (GMFR)	2.18 $\pm$ 1.95	1.68	(0.50, 6.85)	0.19
Whole egg				
6-month	1.19 $\pm$ 1.18	0.86	(0.17, 4.30)	0.57
7-month	0.87 $\pm$ 0.81	0.64	(0.13, 3.02)	0.72
7/6-month (GMFR)	0.83 $\pm$ 0.40	0.74	(0.30, 1.84)	0.74

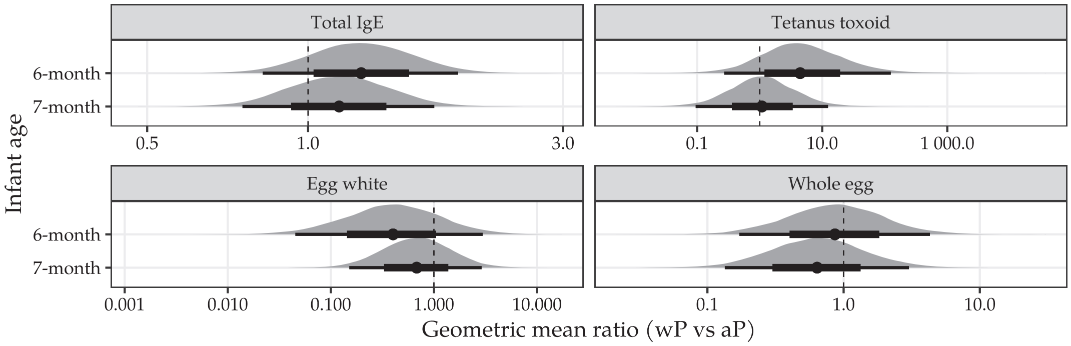

Figure J: Posterior density for geometric mean ratio by vaccination age for total IgE.

D.4 Analyses of IgE Sensitisation

The following sections report on model summaries for analyses of the binary sensitisation outcome defined as IgE concentration ≥ 0.1 kU/L for tetanus-toxoid, egg white, and whole egg IgE.

Table P: Summary of sensitisation model for tetanus toxoid IgE.

	Standardised Pr(IgE ≥ 0.1), Median (95% CrI)				Odds ratios	
	aP	wP	wP − aP	Pr(wP − aP < 0)	Conditional	Marginal
**Tetanus toxoid**						
6-month	0.03 (0.01, 0.08)	0.05 (0.01, 0.12)	0.02 (−0.02, 0.08)	0.18	2.74 (0.31, 31.19)	1.99 (0.46, 13.61)
7-month	0.04 (0.01, 0.10)	0.07 (0.02, 0.15)	0.03 (−0.03, 0.11)	0.18	3.17 (0.24, 52.93)	2.21 (0.37, 17.65)
**Egg white**						
6-month	0.19 (0.10, 0.30)	0.19 (0.10, 0.31)	0.00 (−0.09, 0.10)	0.49	1.03 (0.07, 14.85)	1.01 (0.53, 1.97)
7-month	0.24 (0.14, 0.36)	0.22 (0.12, 0.35)	−0.02 (−0.14, 0.09)	0.65	0.56 (0.03, 10.08)	0.88 (0.42, 1.75)
**Whole egg**						
6-month	0.19 (0.10, 0.31)	0.20 (0.11, 0.32)	0.01 (−0.08, 0.11)	0.41	1.35 (0.10, 20.63)	1.07 (0.57, 2.06)
7-month	0.23 (0.13, 0.35)	0.23 (0.13, 0.36)	0.00 (−0.12, 0.12)	0.52	0.94 (0.04, 20.97)	0.99 (0.48, 1.98)

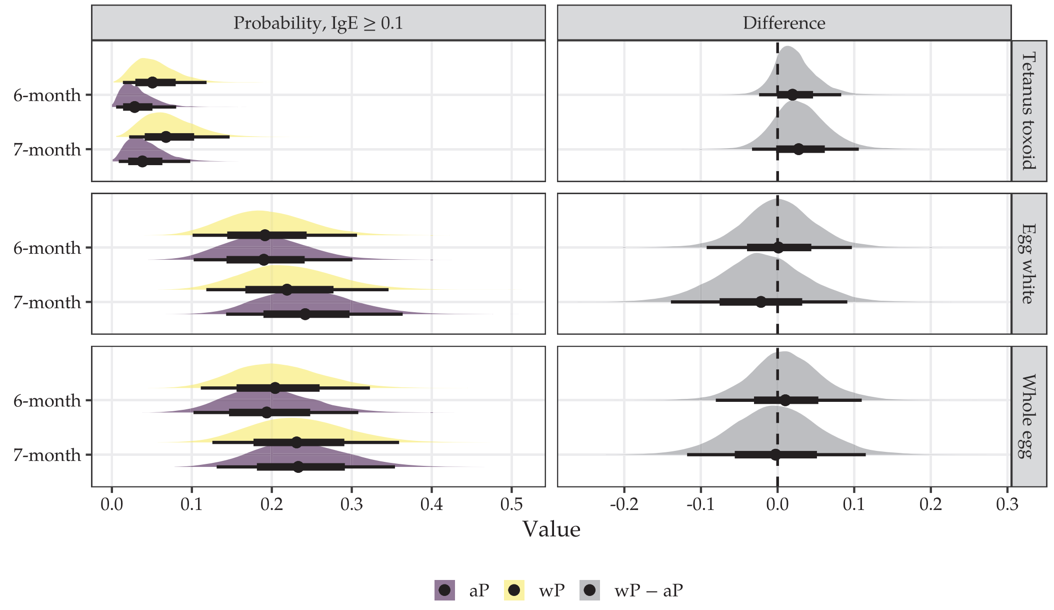

Figure K: Posterior density of standardised probability and difference for IgE sensitisation by age and assigned treatment group.

D.5 Analyses of IgE Quantitation

The following sections report on model summaries for analyses of the binary quantitation outcome defined as IgE concentration ≥ 0.01 kU/L for tetanus-toxoid, egg white, and whole egg IgE.

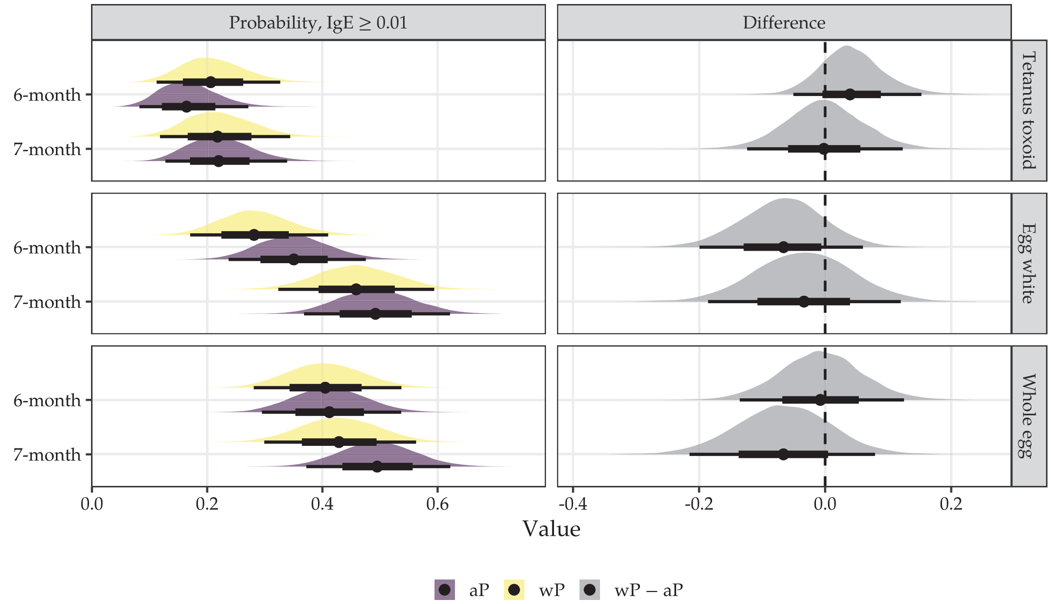

Figure L: Posterior density of standardised probability and difference for IgE quantitation by age and assigned treatment group.

D.6 Sample Summaries (per-protocol)

Table Q: Sample IgE concentration summaries (kU/L) by allergen type and assigned vaccination schedule for per-protocol outcomes.

	aP						wP					
	n	GMC[1]	Q1–Q3	Min–Max	≥0.01	≥0.1	n	GMC[1]	Q1–Q3	Min–Max	≥0.01	≥0.1
Total IgE												
6-month	63	4.83	2.34–10.41	0.40–121.43	100%	100%	59	6.10	2.17–12.87	0.19–141.38	100%	100%
7-month	55	6.55	2.80–11.72	0.52–241.99	100%	100%	51	8.02	2.60–18.02	0.52–148.70	100%	100%
Tetanus-toxoid												
6-month	63	0.00	0.00–0.00	0.00–0.22	13%	2%	59	0.00	0.00–0.00	0.00–1.71	22%	7%
7-month	55	0.00	0.00–0.00	0.00–0.20	22%	2%	51	0.00	0.00–0.00	0.00–0.82	24%	8%
Egg white												
6-month	63	0.00	0.00–0.03	0.00–14.91	35%	19%	59	0.00	0.00–0.01	0.00–3.00	25%	19%
7-month	56	0.00	0.00–0.04	0.00–39.50	52%	23%	51	0.00	0.00–0.03	0.00–4.51	41%	18%
Whole egg												
6-month	63	0.00	0.00–0.03	0.00–14.85	40%	19%	58	0.00	0.00–0.02	0.00–3.51	41%	21%
7-month	56	0.00	0.00–0.04	0.00–33.70	52%	21%	51	0.00	0.00–0.04	0.00–5.16	39%	20%

[1]Geometric mean concentration. Values of 0.00 set to 0.005 for calculation.

D.7 Analyses of IgE Concentrations (per-protocol)

Table R: IgE concentration posterior summaries for Geometric mean ratio (GMR) and geometric mean fold-rise ratio (GMFR), wP vs aP (per-protocol).

	Mean $\pm$ std	Median	95% CrI	Pr(< 1)
Total IgE				
6-month	1.24 $\pm$ 0.29	1.22	(0.78, 1.90)	0.20
7-month	1.05 $\pm$ 0.25	1.03	(0.66, 1.61)	0.45
7/6-month (GMFR)	0.85 $\pm$ 0.11	0.85	(0.66, 1.08)	0.91
Tetanus toxoid				
6-month	19.17 $\pm$ 124.68	4.01	(0.21, 98.89)	0.16
7-month	2.80 $\pm$ 10.44	1.05	(0.07, 14.24)	0.48
7/6-month (GMFR)	0.42 $\pm$ 0.61	0.26	(0.03, 1.79)	0.92
Egg white				
6-month	0.56 $\pm$ 0.87	0.30	(0.02, 2.69)	0.86
7-month	0.78 $\pm$ 0.78	0.55	(0.11, 2.82)	0.76
7/6-month (GMFR)	2.72 $\pm$ 3.47	1.87	(0.45, 10.17)	0.19
Whole egg				
6-month	1.10 $\pm$ 1.18	0.76	(0.13, 4.17)	0.62
7-month	0.74 $\pm$ 0.82	0.52	(0.10, 2.60)	0.79
7/6-month (GMFR)	0.79 $\pm$ 0.44	0.70	(0.24, 1.90)	0.76

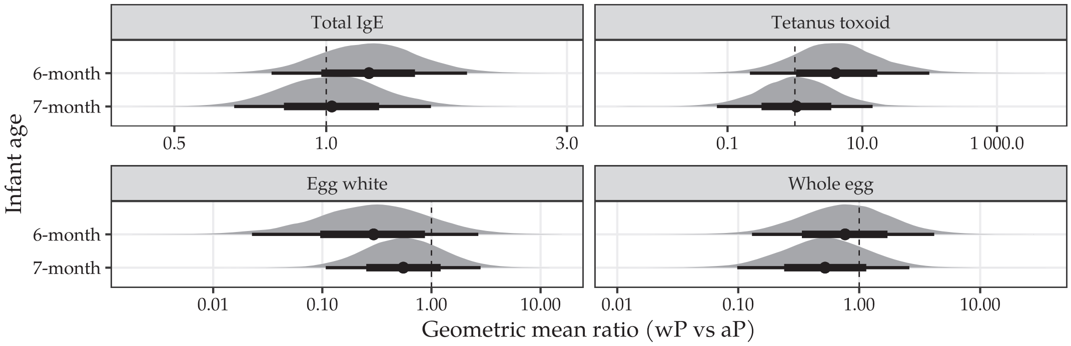

Figure M: Posterior density for geometric mean ratio by vaccination age for total IgE (per-protocol).

D.8 Analyses of IgE Sensitisation (per-protocol)

Table S: Summary of sensitisation model for tetanus toxoid IgE (per-protocol).

	Standardised Pr(IgE ≥ 0.1), Median (95% CrI)				Odds ratios	
	aP	wP	wP − aP	Pr(wP − aP < 0)	Conditional	Marginal
**Tetanus toxoid**						
6-month	0.03 (0.00, 0.08)	0.05 (0.02, 0.13)	0.02 (−0.02, 0.09)	0.16	3.26 (0.32, 49.32)	2.20 (0.49, 18.83)
7-month	0.03 (0.00, 0.09)	0.07 (0.02, 0.15)	0.04 (−0.02, 0.12)	0.12	5.36 (0.32, 131.40)	3.12 (0.47, 35.34)
**Egg white**						
6-month	0.20 (0.11, 0.31)	0.19 (0.10, 0.31)	−0.01 (−0.10, 0.09)	0.55	0.86 (0.06, 11.42)	0.96 (0.49, 1.84)
7-month	0.23 (0.13, 0.36)	0.21 (0.11, 0.34)	−0.02 (−0.14, 0.10)	0.61	0.66 (0.03, 14.10)	0.90 (0.40, 1.90)
**Whole egg**						
6-month	0.20 (0.11, 0.32)	0.21 (0.11, 0.33)	0.00 (−0.09, 0.10)	0.47	1.10 (0.08, 16.01)	1.02 (0.55, 1.95)
7-month	0.22 (0.12, 0.35)	0.23 (0.12, 0.36)	0.00 (−0.11, 0.12)	0.47	1.11 (0.05, 26.73)	1.02 (0.48, 2.15)

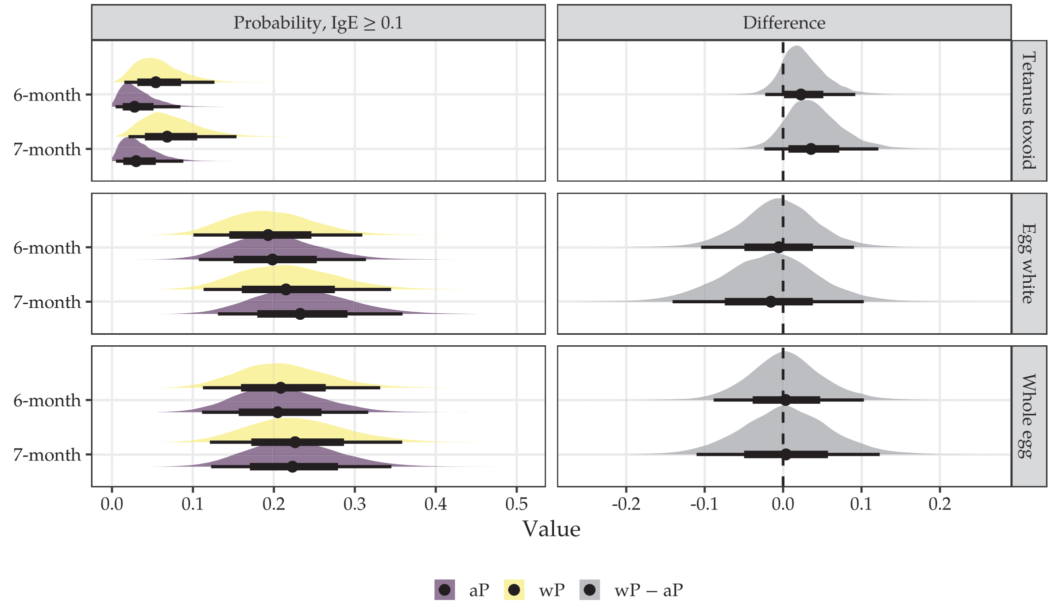

Figure N: Posterior density of standardised probability and difference for IgE sensitisation by age and assigned treatment group (per-protocol).

D.9 Analyses of IgE Quantitation (per-protocol)

Table T: Posterior summaries of quantitation model for IgE concentrations (per-protocol).

	Standardised Pr(IgE ≥ 0.01), Median (95% CrI)				Odds ratios	
	aP	wP	wP − aP	Pr(wP − aP < 0)	Conditional	Marginal
Tetanus toxoid						
6-month	0.18 (0.09, 0.29)	0.22 (0.12, 0.34)	0.04 (−0.06, 0.15)	0.22	2.38 (0.27, 30.73)	1.31 (0.66, 3.01)
7-month	0.24 (0.14, 0.36)	0.24 (0.13, 0.36)	0.00 (−0.13, 0.13)	0.52	0.93 (0.05, 14.57)	0.98 (0.42, 2.29)
Egg white						
6-month	0.35 (0.23, 0.48)	0.27 (0.17, 0.40)	−0.07 (−0.21, 0.05)	0.87	0.34 (0.04, 2.18)	0.69 (0.33, 1.31)
7-month	0.49 (0.36, 0.63)	0.45 (0.32, 0.59)	−0.04 (−0.20, 0.12)	0.69	0.59 (0.07, 5.12)	0.84 (0.40, 1.70)
Whole egg						
6-month	0.41 (0.29, 0.54)	0.41 (0.28, 0.54)	−0.01 (−0.14, 0.13)	0.53	0.92 (0.11, 7.40)	0.98 (0.53, 1.80)
7-month	0.50 (0.37, 0.63)	0.42 (0.29, 0.56)	−0.08 (−0.24, 0.07)	0.85	0.31 (0.03, 3.11)	0.71 (0.34, 1.39)

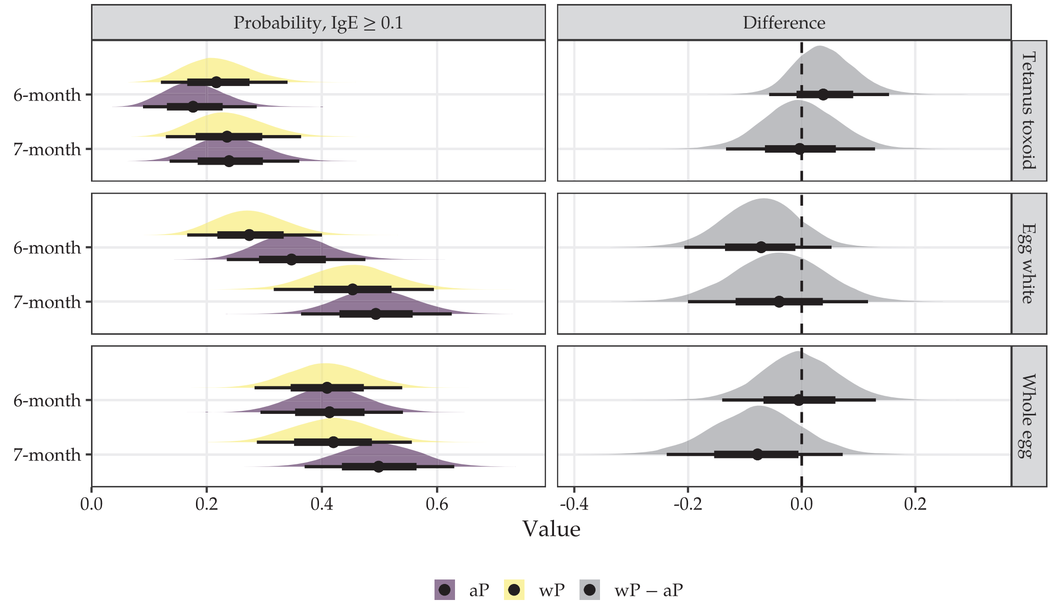

Figure O: Posterior density standardised probability and difference for IgE quantitation by age and assigned treatment group (per-protocol).

E Reactogenicity

E.1 Solicited Systemic Reactions

Table U: Highest intensity grade for systemic reactions following each vaccination (day 0 to 6) by assigned treatment.

	aP (n = 75)					wP (n = 75)				
	0	1	2	3	Missing	0	1	2	3	Missing
6-week										
Irritability	13 (18)	33 (46)	19 (26)	7 (10)	3	9 (12)	28 (38)	26 (35)	11 (15)	1
Vomiting	46 (64)	21 (29)	4 (6)	1 (1)	3	49 (66)	18 (24)	7 (9)	0 (0)	1
Diarrhoea	32 (44)	34 (47)	6 (8)	0 (0)	3	43 (58)	28 (38)	3 (4)	0 (0)	1
Decreased Feeding	53 (74)	16 (22)	3 (4)	0 (0)	3	33 (45)	26 (35)	14 (19)	1 (1)	1
Drowsiness	32 (45)	33 (46)	6 (8)	0 (0)	4	18 (24)	38 (51)	15 (20)	3 (4)	1
Restlessness	20 (28)	32 (44)	14 (19)	6 (8)	3	21 (28)	26 (35)	20 (27)	7 (9)	1
4-month										
Irritability	16 (22)	25 (34)	24 (33)	8 (11)	2	18 (25)	29 (41)	16 (23)	8 (11)	4
Vomiting	58 (79)	12 (16)	3 (4)	0 (0)	2	53 (75)	16 (23)	2 (3)	0 (0)	4
Diarrhoea	52 (71)	17 (23)	4 (5)	0 (0)	2	51 (72)	19 (27)	1 (1)	0 (0)	4
Decreased Feeding	52 (71)	18 (25)	3 (4)	0 (0)	2	51 (72)	18 (25)	1 (1)	1 (1)	4
Drowsiness	42 (58)	20 (27)	8 (11)	3 (4)	2	33 (46)	34 (48)	4 (6)	0 (0)	4
Restlessness	31 (42)	27 (37)	11 (15)	4 (5)	2	30 (42)	31 (44)	7 (10)	3 (4)	4
6-month										
Irritability	25 (37)	25 (37)	14 (21)	4 (6)	7	28 (41)	29 (42)	7 (10)	5 (7)	6
Vomiting	56 (82)	11 (16)	1 (1)	0 (0)	7	58 (84)	10 (14)	1 (1)	0 (0)	6
Diarrhoea	60 (88)	5 (7)	3 (4)	0 (0)	7	66 (96)	2 (3)	1 (1)	0 (0)	6
Decreased Feeding	51 (75)	13 (19)	4 (6)	0 (0)	7	54 (78)	13 (19)	2 (3)	0 (0)	6
Drowsiness	48 (71)	18 (26)	2 (3)	0 (0)	7	52 (75)	17 (25)	0 (0)	0 (0)	6
Restlessness	37 (54)	22 (32)	7 (10)	2 (3)	7	47 (68)	16 (23)	6 (9)	0 (0)	6

For participants with non-responses for some days, the maximum size is taken as the largest size reported on days with responses available. Percentages exclude participants with a missing highest grade (diary card not returned, or non-response for all days).

Table V: Experience of any severe intensity systemic reaction following vaccination (day 0 to 6) by assigned treatment.

	aP (n = 75)	wP (n = 75)
6-week	8/72 (11%)	14/74 (19%)
4-month	12/73 (16%)	9/71 (13%)
6-month	5/68 (7%)	5/69 (7%)

E.1.1 Irritability

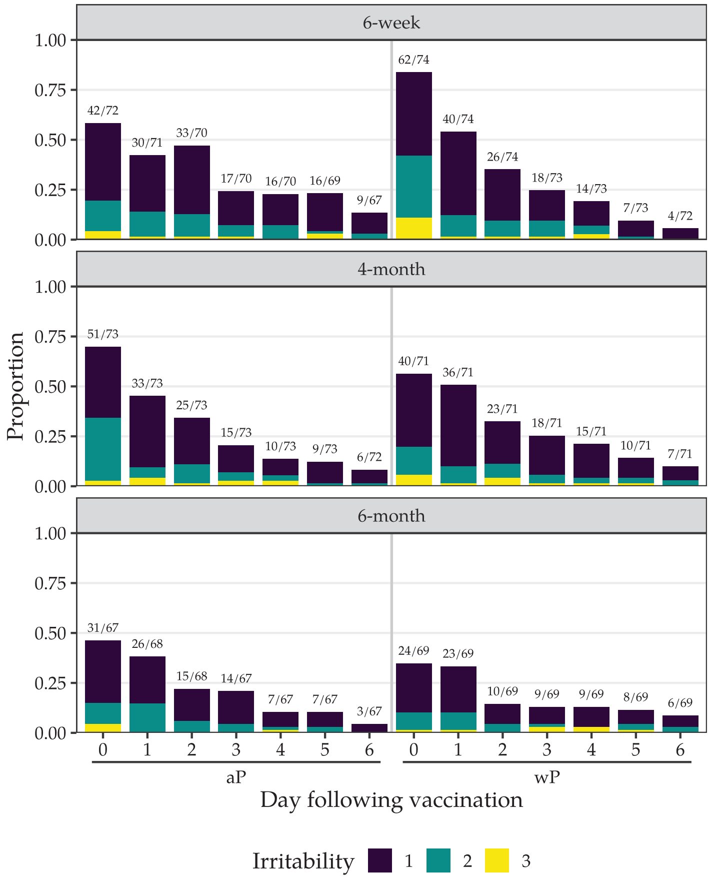

Figure P: Irritability daily intensity grades, day 0 to 6 by vaccination age and assigned treatment.

E.1.2 Vomiting

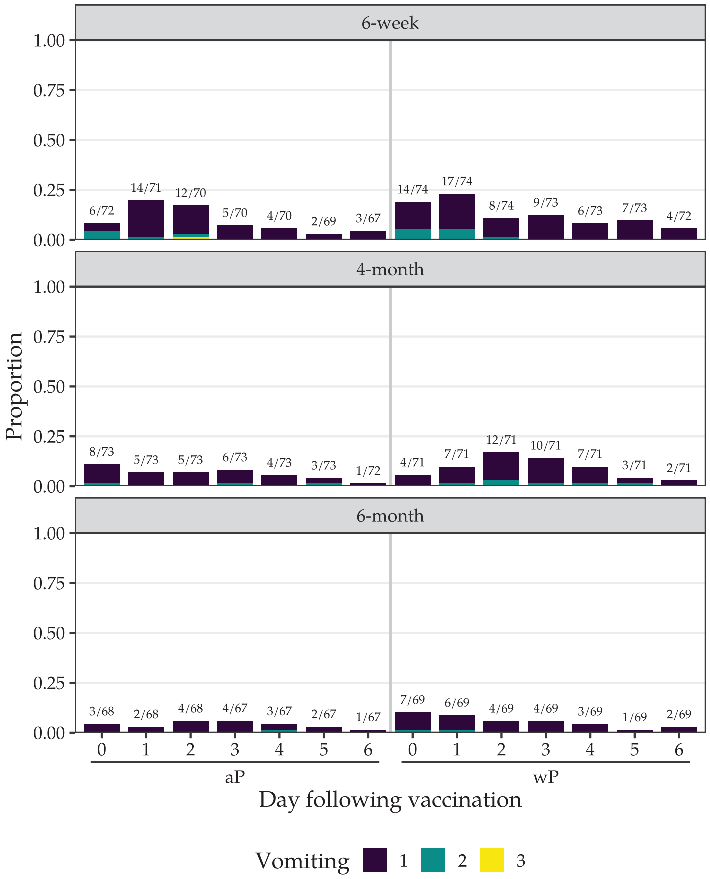

Figure Q: Vomiting daily intensity grades, day 0 to 6 by vaccination age and assigned treatment.

E.1.3 Diarrhoea

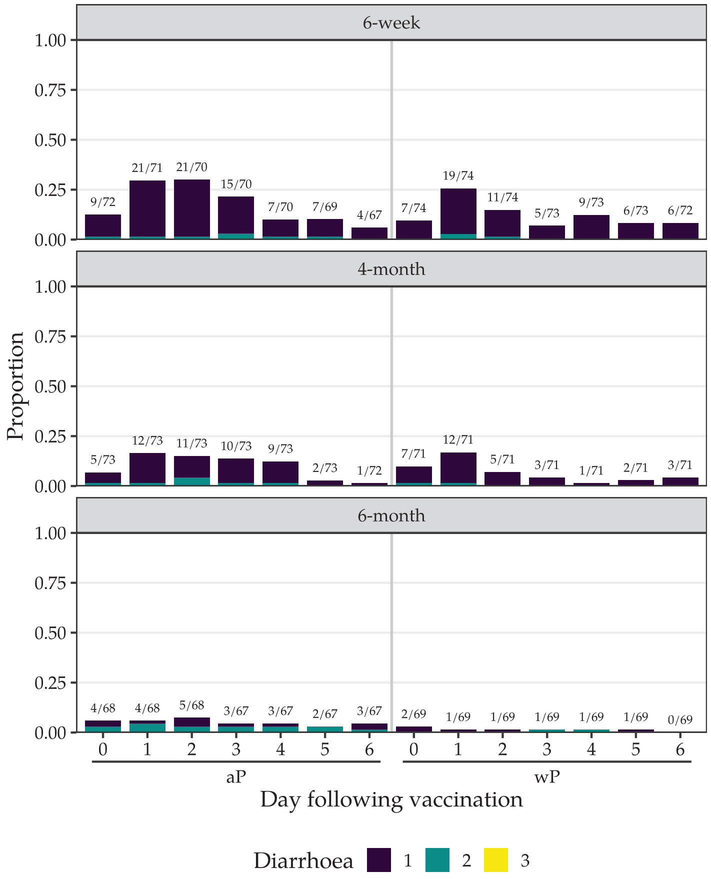

Figure R: Diarrhoea daily intensity grades, day 0 to 6 by vaccination age and assigned treatment.

E.1.4 Decreased Feeding

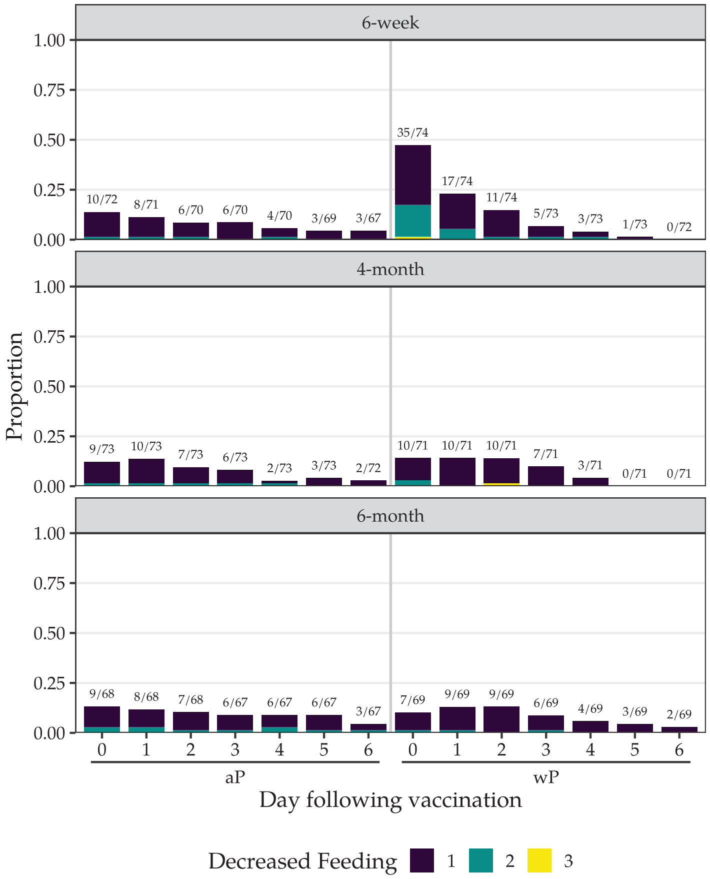

Figure S: Decreased feeding daily intensity grades, day 0 to 6 by vaccination age and assigned treatment.

E.1.5 Drowsiness

Figure T presents the distribution of daily intensity grades for drowsiness following each vaccination occassion by assigned treatment.

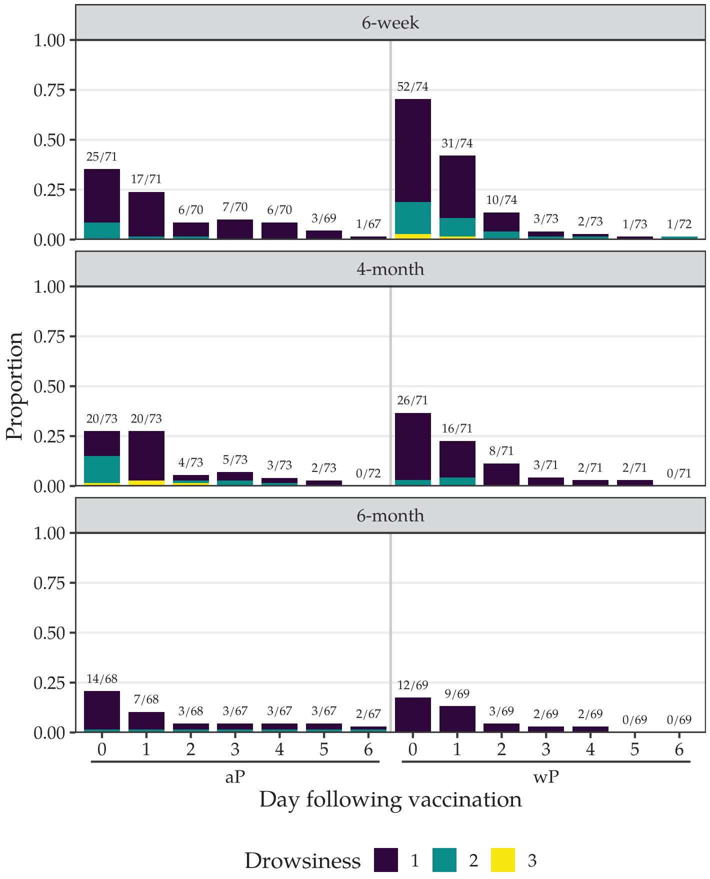

Figure T: Drowsiness daily intensity grades, day 0 to 6 by vaccination age and assigned treatment.

E.1.6 Restlessness

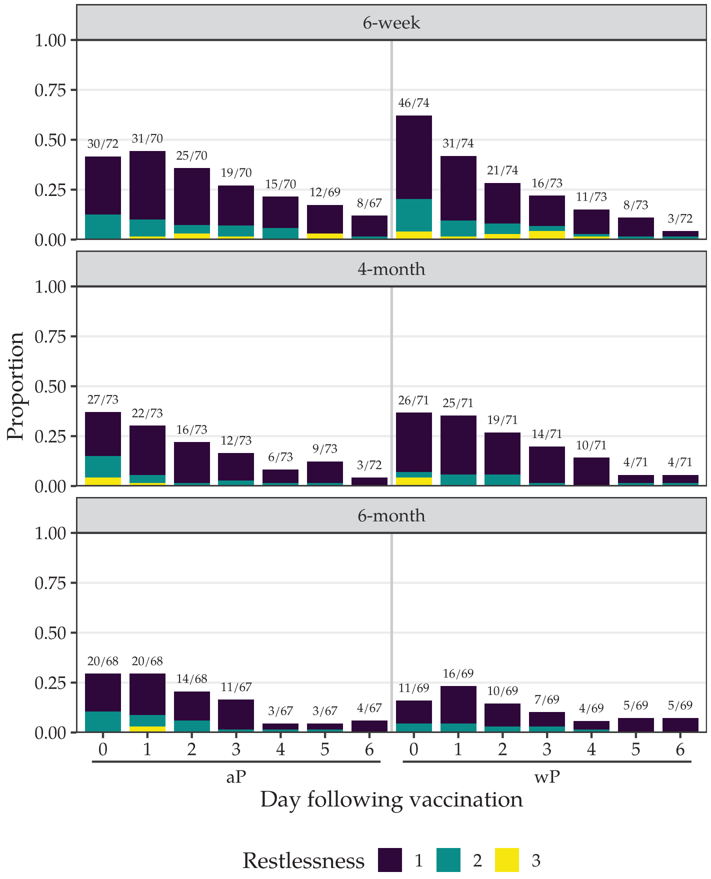

Figure U: Restlessness daily intensity grades, day 0 to 6 by vaccination age and assigned treatment.

E.1.7 Fever

Table W: Highest fever following each vaccination by assigned treatment.

	aP (n = 75)				wP (n = 75)		
	None (<38)	38-38.4	38.5-38.9	Missing	None (<38)	38-38.4	Missing
6-week	69 (99)	1 (1)	0 (0)	5	73 (100)	0 (0)	2
4-month	68 (94)	3 (4)	1 (1)	3	67 (96)	3 (4)	5
6-month	67 (99)	1 (1)	0 (0)	7	67 (99)	1 (1)	7

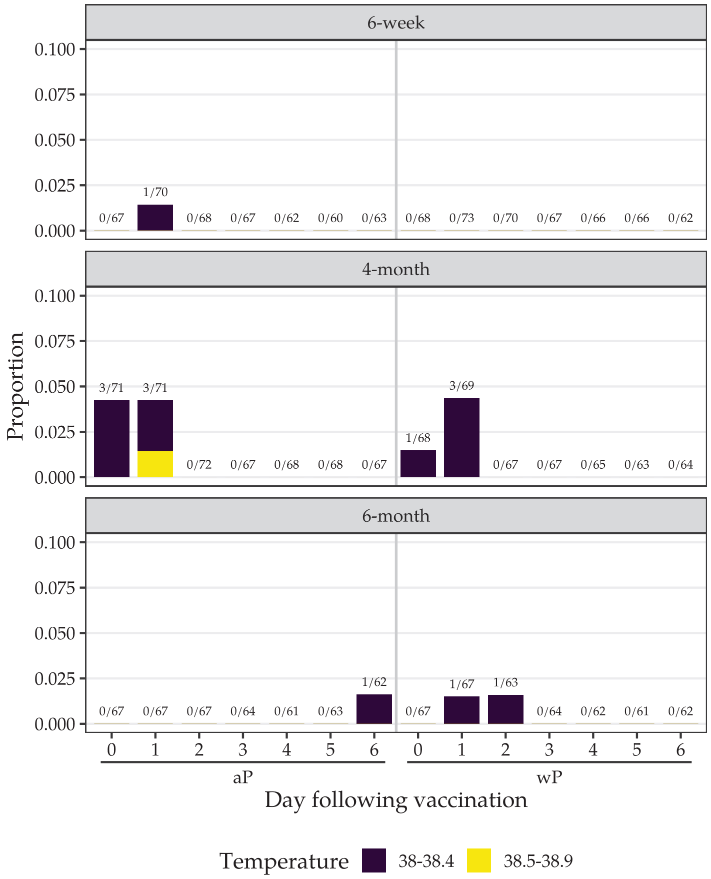

Figure V: Fever day 0 to 6 by vaccination age and assigned treatment.

E.2 Solicited Injection Site Reactions

Table X: Maximum injection site reaction size in the 7 days following vaccination, by vaccination age, vaccine, and assigned treatment.

	Size (mm)											
	aP (n = 75)						wP (n = 75)					
	None	>0 to 10	>10 to 25	>25 to 50	>50	Missing	None	>0 to 10	>10 to 25	>25 to 50	>50	Missing
**6-week - aP or wP**												
Erythema	50 (69)	22 (31)	0 (0)	0 (0)	0 (0)	3	36 (49)	36 (49)	0 (0)	1 (1)	1 (1)	1
Swelling	64 (89)	7 (10)	1 (1)	0 (0)	0 (0)	3	50 (68)	19 (26)	2 (3)	2 (3)	1 (1)	1
Induration	60 (83)	11 (15)	1 (1)	0 (0)	0 (0)	3	37 (50)	27 (36)	6 (8)	4 (5)	0 (0)	1
**6-week - 13vPCV**												
Erythema	51 (71)	20 (28)	1 (1)	0 (0)	0 (0)	3	45 (61)	27 (36)	1 (1)	0 (0)	1 (1)	1
Swelling	66 (92)	6 (8)	0 (0)	0 (0)	0 (0)	3	58 (78)	12 (16)	3 (4)	0 (0)	1 (1)	1
Induration	54 (75)	18 (25)	0 (0)	0 (0)	0 (0)	3	51 (69)	18 (24)	5 (7)	0 (0)	0 (0)	1
**4-month - DTaP-Hib-HepB-IPV**												
Erythema	36 (49)	30 (41)	6 (8)	1 (1)	0 (0)	2	40 (56)	28 (39)	2 (3)	1 (1)	0 (0)	4
Swelling	53 (73)	15 (21)	2 (3)	3 (4)	0 (0)	2	62 (87)	8 (11)	0 (0)	1 (1)	0 (0)	4
Induration	44 (60)	23 (32)	3 (4)	3 (4)	0 (0)	2	54 (76)	14 (20)	2 (3)	1 (1)	0 (0)	4
**4-month - 13vPCV**												
Erythema	39 (53)	28 (38)	5 (7)	1 (1)	0 (0)	2	39 (55)	29 (41)	2 (3)	1 (1)	0 (0)	4
Swelling	52 (71)	17 (23)	2 (3)	2 (3)	0 (0)	2	63 (89)	6 (8)	1 (1)	1 (1)	0 (0)	4
Induration	47 (64)	20 (27)	5 (7)	1 (1)	0 (0)	2	54 (76)	14 (20)	1 (1)	2 (3)	0 (0)	4
**6-month - DTaP-Hib-HepB-IPV**												
Erythema	26 (39)	36 (54)	4 (6)	1 (1)	0 (0)	8	28 (41)	30 (44)	9 (13)	1 (1)	0 (0)	7
Swelling	50 (75)	14 (21)	2 (3)	1 (1)	0 (0)	8	53 (78)	9 (13)	3 (4)	3 (4)	0 (0)	7
Induration	37 (55)	28 (42)	2 (3)	0 (0)	0 (0)	8	34 (50)	24 (35)	7 (10)	3 (4)	0 (0)	7

For participants with non-responses for some days, the maximum size is taken as the largest size reported on days with responses available. Percentages exclude participants with a missing highest grade (diary card not returned, or non-response for all days).

E.2.1 Erythema

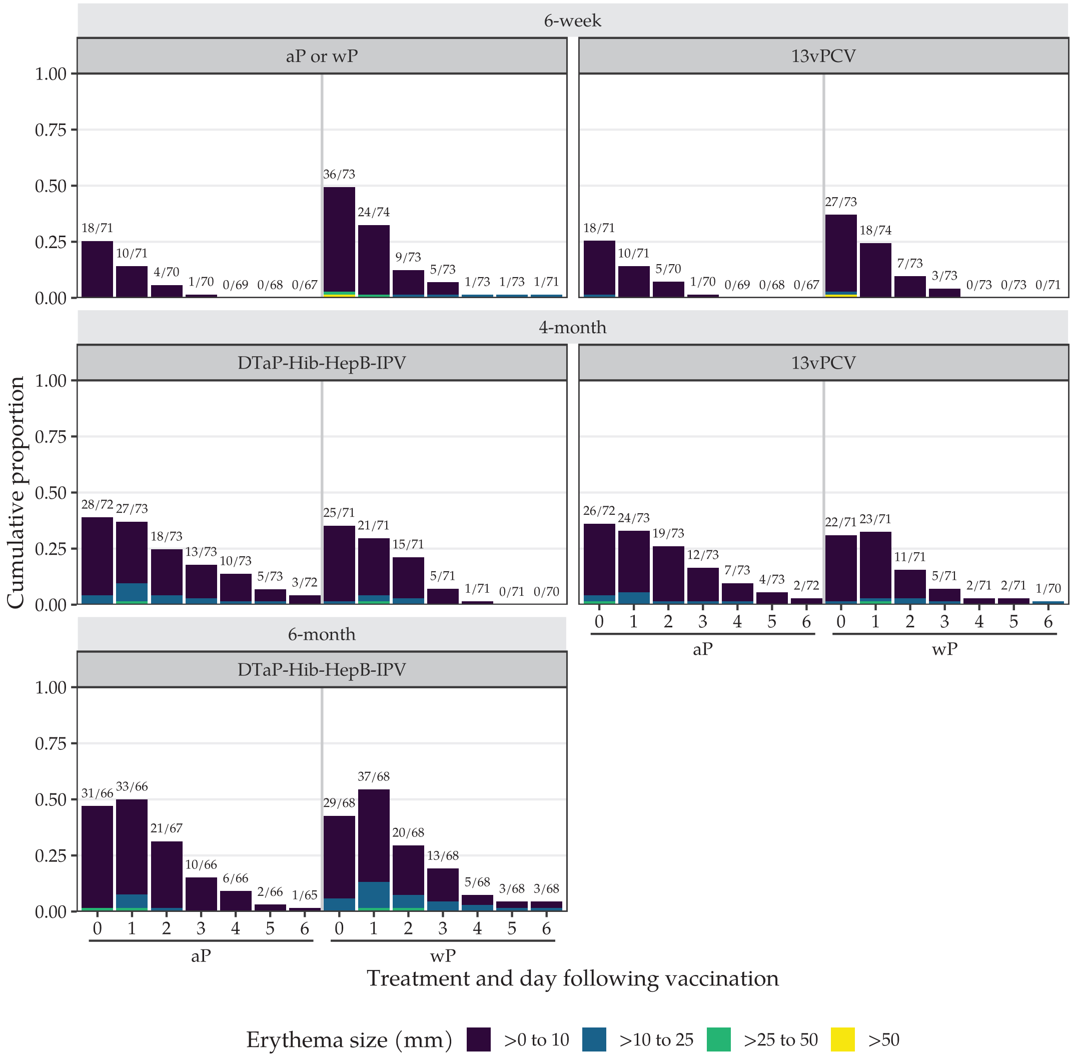

Figure W: Injection site erythema by vaccination age, vaccine, and assigned treatment.

E.2.2 Swelling

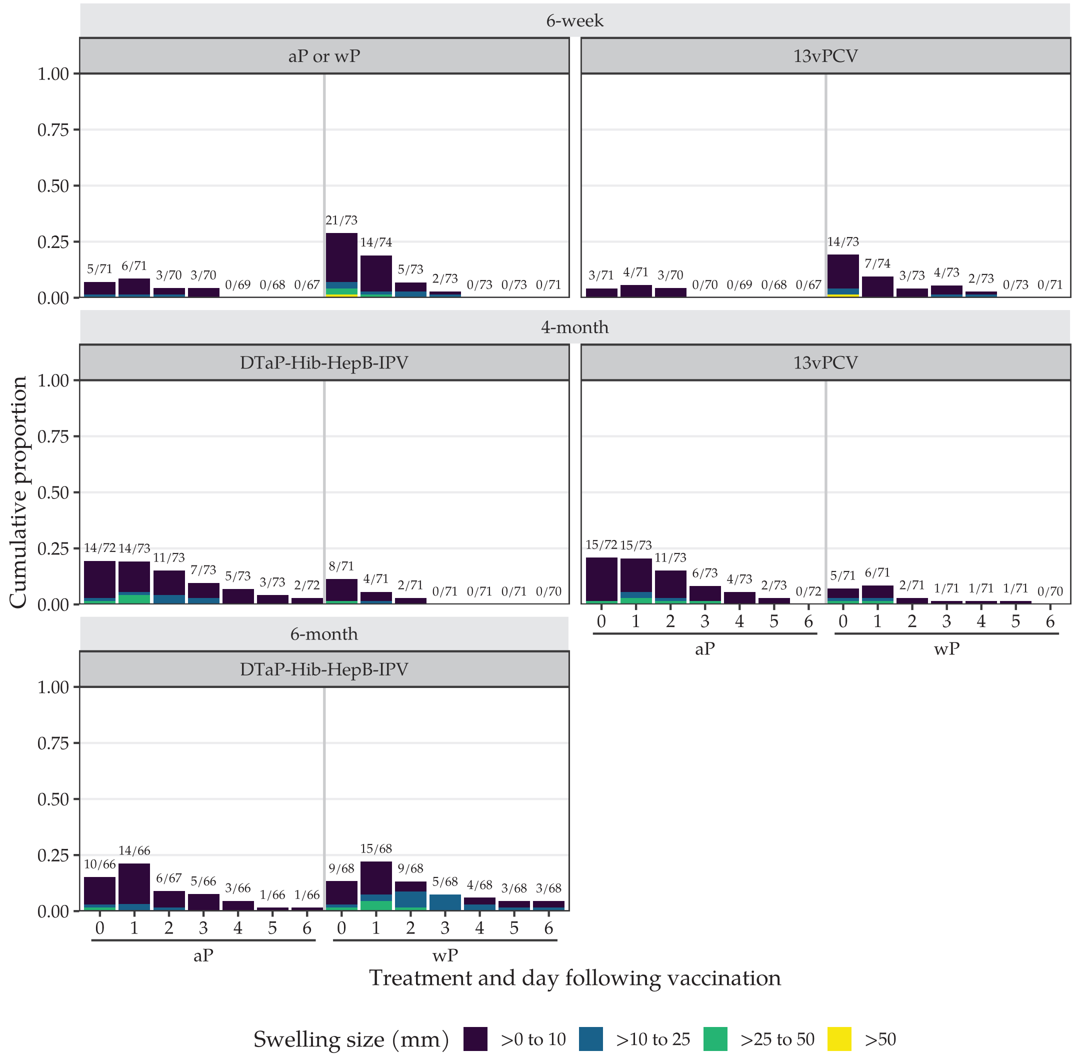

Figure X: Injection site swelling by vaccination age, vaccine, and assigned treatment.

E.2.3 Induration

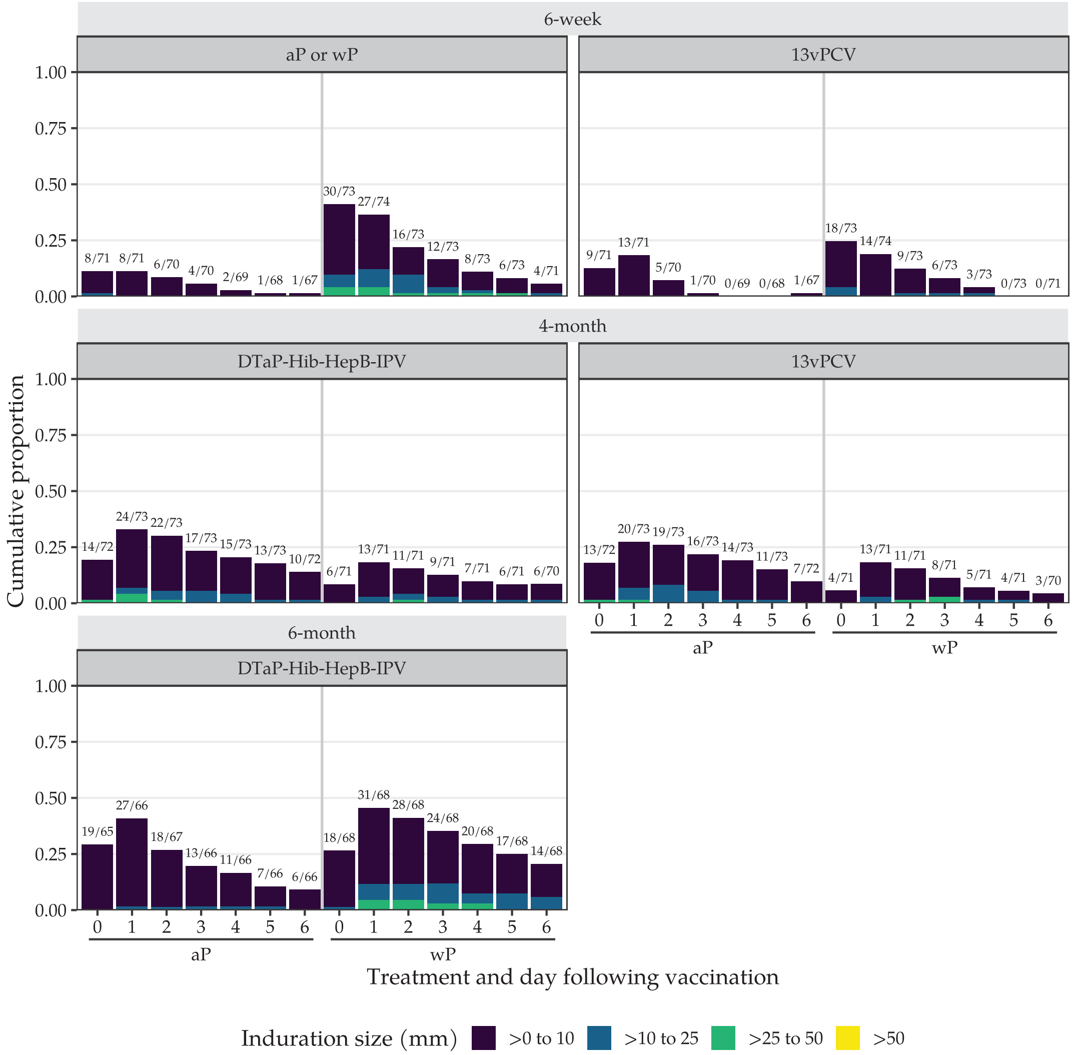

Figure Y: Injection site induration by vaccination age, vaccine, and assigned treatment.

E.2.4 Pain

Table Y: Maximum injection site pain in the 7 days following vaccination, by vaccination age, vaccine, and assigned treatment.

	Size (mm)									
	aP (n = 75)					wP (n = 75)				
	None	Mild	Moderate	Severe	Missing	None	Mild	Moderate	Severe	Missing
6-week										
aP or wP	54 (75)	15 (21)	3 (4)	0 (0)	3	30 (41)	21 (28)	17 (23)	6 (8)	1
13vPCV	52 (72)	18 (25)	1 (1)	1 (1)	3	38 (51)	21 (28)	13 (18)	2 (3)	1
4-month										
DTaP-Hib-HepB-IPV	55 (75)	11 (15)	6 (8)	1 (1)	2	52 (73)	14 (20)	3 (4)	2 (3)	4
13vPCV	52 (71)	12 (16)	8 (11)	1 (1)	2	52 (73)	15 (21)	2 (3)	2 (3)	4
6-month										
DTaP-Hib-HepB-IPV	49 (73)	13 (19)	3 (4)	2 (3)	8	42 (62)	23 (34)	3 (4)	0 (0)	7

For participants with non-responses for some days, the maximum grade is taken as the highest grade reported on days with responses available. Percentages exclude participants with a missing highest grade (diary card not returned, or non-response for all days).

E.3 Parental Satisfaction

Table Z: Number and percentage of respondents by response to the statement: "I would be willing to have another child have this combination of immunisations at this age."

	aP (n = 75)	wP (n = 75)
Completed		
Strongly disagree	0 (0%)	0 (0%)
Disagree	0 (0%)	0 (0%)
Neither agree nor disagree	3 (4%)	2 (3%)
Agree	13 (18%)	24 (33%)
Strongly agree	56 (78%)	47 (64%)
Not completed		
Not completed	3 (4%)	2 (3%)

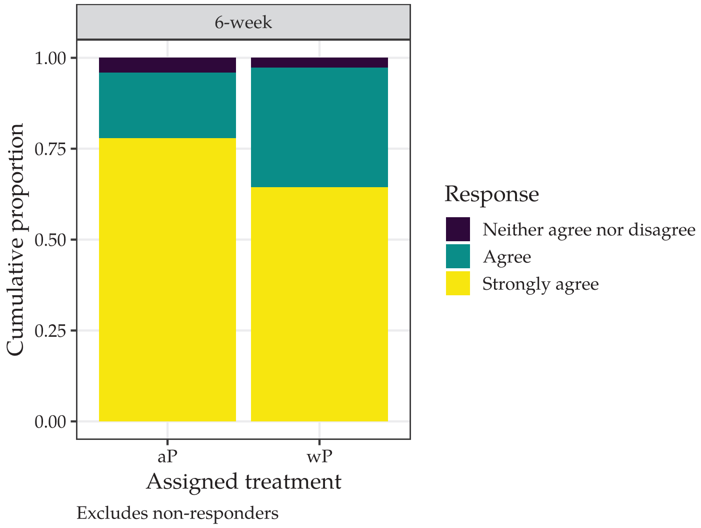

Figure Z: Distribution of responses to "I would be willing to have another child have this combination of immunisations at this age" by vaccination occassion and assigned treatment.

References

Hedeker, Donald, Stephen H. C. du Toit, Hakan Demirtas, and Robert D. Gibbons. 2018. "A Note on Marginalization of Regression Parameters from Mixed Models of Binary Outcomes." *Biometrics* 74 (1): 354–61.

Totterdell, James A., Gladymar Perez Chacon, Marie J. Estcourt, Mark Jones, Peter Richmond, Thomas L. Snelling, and Julie A. Marsh. 2022. "Statistical Analysis Plan for the OPTIMUM Study: Optimising Immunisation Using Mixed Schedules, an Adaptive Randomised Controlled Trial of a Mixed Whole-Cell/Acellular Pertussis Vaccine Schedule." *Trials* 23 (1).

Wiley, Joshua F., and Donald Hedeker. 2022. *brmsmargins: Bayesian Marginal Effects for 'brms' Models*.
