# Supplementary material for: Immunogenicity, reactogenicity, and IgE-mediated immune responses of a mixed whole-cell and acellular pertussis vaccine schedule in Australian infants: A randomised, double-blind, noninferiority trial
Source: PLoS Med. 2024 Jun 10;21(6):e1004414. doi: 10.1371/journal.pmed.1004414 (PMC11198910; doi:10.1371/journal.pmed.1004414)
Supplement: Attachment — Submitted filename: 13-04_6m_plos_med_revisions_v2.0.docx [file pmed.1004414.s003.docx]

| **Reviewer #1 comments** [Numbering added by editor] | **Authors’ answers** |
| --- | --- |
| In this study the authors report the safety and immunogenicity data from a RCT that investigates the effect of the primary dose of pertussis vaccination. The study is generally very well executed and addresses an interesting and important concept, i.e. that different pertussis vaccine types with known distinct effects on immunological imprinting against pertussis antigens may also have off-target effects, such as IgE-mediated food allergy. Even though no differences were observed with regards to the IgE measurements in the current smaller study, it will be very interesting to see what happens in the larger cohort. | We thank the reviewer for their feedback and appreciate the time spent reviewing our manuscript. |
| 1. Whilst the study and reported endpoints are clearly described, please find below a number of comments/questions that the authors may wish to address: |  |
| 2. The authors focus on the comparison between the acellular and the whole cell pertussis vaccine. However, given that the pentavalent whole cell vaccine does not contain IPV and the hexavalent acellular vaccine does, and that a standalone dose of IPV was not administered together with the pentavalent wP at 2m of age but rather at the age of 18m (i.e. after the immunological endpoints), the authors should discuss the potential role of IPV as it cannot be formally ruled out that it also plays a role. | **Agree.** The following comment has been added to the limitations (discussion section, lines 471 to 475). *Apart from the difference between regimens in wP content, there wero also differences in the content of IPV (absent in Pentabio PT Bio Farma), aluminium (lower in Pentabio PT Bio Farma), and adventitial antigens.[16] We can therefore not be certain whether any observed differences between regimens can be attributed to the difference in the wP content or to these other factors.* |
| 3. Based on the methods section I assume that the assay was run blinded and randomized. Were the samples individually paired on the same run/batch? How was QC done in the immunological assays? And what was the reproducibility of the assays? This is particularly relevant for the IgE measurements that seem less standardized (I may be wrong). | a. Immunological assays were run blinded and randomised. b. QC for the IgG assays followed the protocol developed by McAlister and colleagues (PMID: 34430260, PMID: 33589299). c. QC for the IgE assays followed standardised SOPs developed by PathWest pathology lab (Department of Health, Western Australia, Australia). d. Reproducibility of the assays.  - Multiplex fluorescent bead-based immunoassay: “less than 10% coefficient of variance (CV) was calculated for all antigens, except FIM 2/3 which was <12%, indicating good assay reproducibility” (PMID: 34430260). - ImmunoCAP: “low CV, good precision and reproducibility and reports down to 0.1 kUA/L (range, 0.1-100 kUA/L”; PMID: 28552762). Review of end of cycle testing on Royal College of Pathology Australasian Quality Assurance programme shows an SD of 24.5 with a CV of 5.1% for total IgE.   The methods section has been amended as follows (lines 185 to 201):  Peripheral blood samples were obtained immediately before the 6-month aP dose, and approximately one month afterwards. *All immunological assays were performed blinded.* Serum IgG responses to diphtheria, tetanus, and pertussis antigens were measured using a multiplex fluorescent bead-based immunoassay. *The assay’s operating characteristics and quality control procedures are described elsewhere.[21,22]* The threshold for seropositivity was defined as concentrations ≥ 5 IU/mL (5000 mIU/mL) for pertussis antigens (i.e., PT, FHA, pertactin [PRN], and FIM2/3).[23] Long-term seroprotective concentrations for TT and DT were defined at ≥ 100 mIU/mL. [24,25]  Total and specific IgE to hen’s egg antigens and TT were measured in plasma by means of the *ImmunoCAP assay (Thermo Fisher Scientific)* using the limits of detection (LLoD) and quantitation (LLoQ) specified by the manufacturer. *The analyses were performed at PathWest (Department of Health, Western Australia), under standardised study operating procedures. The assay’s operating characteristics are described elsewhere.[26–28]* The total IgE concentrations were reported in the range of 0·1 kU/L to 100 kU/L for low-range assays or 2 to 5000 kU/L if exceeded *(the accuracy of the immunoassay within ± 8% of the WHO IgE reference value with a 95% probability; trueness of the calibrator within ± 2% compared to the WHO IgE reference value with a 95% probability)*.[27] IgE concentrations to specific antigens were reported in the range of 0·00 kU_a_/L to 100·00 kU_a_/L.[28] The LLoD for antigen/allergen specific IgE was ≥ 0·01 kU/L; the LLoQ for total and specific IgE was ≥ 0·1 kU_(a)_/L. [28] |
| 4. It is not always very clearly described for each modeled immune response analysis in the main/supplementary data which parameters are included. | **Noted.** This information was included in the original manuscript (Statistical analysis: lines 197 and 198 🡪 “All adjusted models included sex, birth order, breastfeeding status, delivery method, family history of atopic disease, and parental income as baseline covariates).” The following sentence was included in the results section (lines 264 to 266; lines 300 to 301) and in section 1 of the supplementary document: “Results from unadjusted models were generally consistent with the adjusted models, therefore, results from unadjusted models are not reported.” |
| 5. The authors report higher solicited systemic AEs in the wP-group compared to the aP-group (lines 307-310) at 6w of age but not after the subsequent doses. Were AEs reported at the different time points observed in the same infants or were these different infants? | **Noted and added to the results section (lines 361 to 363).** *Four infants assigned to the aP-only schedule and five infants assigned to the mixed schedule experienced grade 3 solicited systemic adverse reactions on two occasions. No severe recurrent AEs were reported in the remaining infants*.  As a complete breakdown, let “xxx” denote event occurrence after the 6-week, 4-month, and 6-month doses, e.g. “011” means no event reported after the 6-week dose, but events reported after the 4-month and 6-month doses:   - aP: 2 - 011, 2 - 110 - wP: 2- 011, 2 - 110, 1 - 101. |
| 6. Figures 2/3 in the main paper show the antibody responses at the 6 and 7m time points. It would be helpful if these figures would also show whether differences between groups/time points are statistically different or not. | **Noted.** Because of the multiplicity of tests, traditional significance testing is likely to be misleading or highly conservative. Instead, we provide an estimate of the effect of the intervention (wP vs aP) on the immunological outcomes of interest along with 95% credible intervals at each time point, as described in Tables 2 and 3. |

| **Reviewer #2 comments** [Numbering added by editor] | **Authors’ response** |
| --- | --- |
| In this study, Gladymar Pérez Chacón and colleagues analyzed the immunogenicity and reactogenicity of a mixed pertussis vaccination schedule in a randomized double-blind clinical trial. The novelty of this study resides in the direct comparison of the effect of a whole-cell pertussis vaccine administered as the first priming vaccination compared to an acellular pertussis vaccine. The aim was to understand not only the implications of reactogenicity and immunogenicity but also the IgE-mediated immune responses and possible associations of acellular pertussis vaccines with the development of IgE-mediated food allergies. Although this trial is well designed and conducted, the results of this report only concern stage 1, which reports relatively fewer individuals and short time points and do not include stage 2, which encompasses a later time point. However, stage 2 addresses a much more important question and covers more individuals by a large margin. Nevertheless, I believe the manuscript is of interest and could be improved by addressing the suggestions below. | We thank the reviewer for their feedback and appreciate the time spent reviewing our manuscript. We acknowledge that the stage two results will be of interest, but given the anticipated sample-size and follow-up, the stage two results are unlikely to be available for several years. Here we report the stage one results in the interest of expeditious reporting. |
| General comments:  1. My primary concern is the fact that the findings on IgE reactivity are fairly inconclusive since they only pertain to stage 1 of the trial. The study would be much more interesting if data from stage 2 were combined with the present manuscript. Alternatively, a manuscript solely focused on reactogenicity and immunogenicity would still address a solid concern associated with the mixed vaccine schedule and would not leave the reader wondering if an effect of IgE could or could not be observed in the next chapter. | We acknowledge that the IgE data are not conclusive and that the reactogenicity and immunogenicity results are of greater interest. We are not collecting any further IgE results in stage two, so we believe, in the interest of transparency, it is most appropriate to report all available stage one data as planned (i.e., reactogenicity, immunogenicity, IgE-mediated immune responses, and parental acceptability), ahead of primary clinical outcome data (IgE-mediated food allergy by 12 months old). |
| 2. Although the authors acknowledge that T cell responses are important and will be evaluated, no immunogenicity data is present in the current manuscript. I believe that this data combined with serology would be much more informative and interesting for the readership of this Journal. | We acknowledge that there will be interest in T-cell responses which were not pre-specified in the stage one protocol. In late 2023 the investigators secured funding to conduct these experiments, which will be evaluated as secondary endpoints in a subcohort of infants enrolled in stage one (e.g., those who provided consent to have blood samples collected ~ 72 h after the 6-month dose of aP). As these were not pre-specified in the stage one protocol, we propose not to delay publication of the stage one results for their inclusion. |
| Specific comments: 3. Abstract: Both findings and conclusions do not mention IgE responses, which would be expected since it is stated in the title. As per my suggestions above, I would remove entirely this topic from this manuscript since I don't think Stage 1 is equipped to answer what I think is one of the main and most important goals of this study. [Editors’ note: while we appreciate the opinion of the reviewer, the outcomes should be reported as prespecified in the study protocol.] | We are guided by both the editor’s note and the reviewer’s comment. IgE data have now been included in the findings and conclusions sections of the abstract (lines 43 to 70). The edits are presented below:  **Methods**  OPTIMUM is a Bayesian, two-stage, double-blind, randomised trial. At 6 weeks old, infants are assigned (1:1) to either a first dose of a pentavalent wP combination vaccine (DTwP-Hib-HepB, *Pentabio PT Bio Farma, Indonesia*) or a hexavalent aP vaccine (DTaP-Hib-HepB-IPV, *Infanrix Hexa, GlaxoSmithKline, Australia*). All infants receive the hexavalent aP vaccine at 4 and 6 months old as well as an aP vaccine at 18 months old (DTaP-IPV, Infanrix-IPV, *GlaxoSmithKline, Australia). Serum IgG responses to diphtheria, tetanus, and pertussis antigens were measured using a multiplex fluorescent bead-based immunoassay; total and specific IgE were measured in plasma by means of the ImmunoCAP assay (Thermo Fisher Scientific).* The immunogenicity of the mixed schedule was defined as being non-inferior to that of the aP-only schedule using a non-inferiority margin of 2/3 on the ratio of the geometric mean concentrations (GMR) of pertussis toxin (PT)-IgG one month after the 6-month aP dose. Solicited adverse reactions were summarised by study arm and included all children who received the first dose of either wP or aP. Parental acceptance was assessed using a five-point Likert scale. The trial is registered with ANZCTR (ACTRN12617000065392p).  **Findings**  Between March 7, 2018 and January 13, 2020, 150 infants were randomised (75 per arm). PT-IgG responses of the mixed schedule were non-inferior to the aP-only schedule at approximately one month after the 6-month aP dose [GMR = 0·98, 95% credible interval (0·77–1·26); probability (GMR>2/3)>0·99; intent-to-treat analysis]. *At 7 months old, the posterior median probability of quantitation for tetanus toxoid IgE was 0·22 (95% credible interval 0·12, 0·34) in both, the mixed schedule group and in the aP-only group.* At 6 weeks old, severe systemic reactions were reported among 14/74 (19%) infants after wP and 8/72(11%) infants after aP. Parental acceptance of mixed and aP-only schedules was high (97% versus 96% would agree to have the same schedule again).  **Conclusions**  Compared to the aP-only schedule, the mixed schedule evoked non-inferior PT-IgG responses, was associated with more severe reactions, but was well accepted by parents. Tetanus toxoid IgE responses did not differ across the study groups. |
| Introduction:  4. Line 76 - References are more than 10 years old. I am wondering if there are more compelling and recent evidences. | **Noted.** The cited papers describe pertussis epidemics in the states of Queensland, Australia (2009) and Oregon (US, 2012). Both illustrate that a high proportion of pertussis notifications occurred in the first birth cohorts who received a first dose of aP in place of wP. The authors are unaware of more recent publications on the same topic. This is perhaps explained by the fact that the ‘natural experiment’ of transition from wP to aP occurred in the USA and many similar countries (including Australia) almost 30 years ago. |
| 5. Line 83 - When discussing the impact on food allergy, it would be interesting also to note that similar studies showed no differences in childhood asthma (PMID: 37792889). Also, it would be interesting to point out that two other studies showed differential expression of IgE in aP- vs wP-primed cohorts for either pertussis or non-pertussis vaccine antigens (PMID: 31419322, PMID: 33690224). | **Noted.** Findings from PMID: 37792889 (Pérez Chacón – Snelling’s Lab, 2023) have been added to the introduction (lines 111 to 113). Findings from PMID: 33690224 (da Silva Antunes – Sette’s/Peters’ Lab, 2021) were initially referenced in the discussion section of the original submission and have now been incorporated into the introduction as well (lines 119 to 122). Findings from PMID: 31419322 (Aalberse, 2019) had already been cited in the original submission as Ref 10. The edits are presented below:  We have previously reported that compared to matched population controls, Australian children with IgE-mediated food allergy were less likely to have received wP rather than aP as their first pertussis vaccine dose in infancy (OR 0·77; 95% CI 0·62–0·95).[5] In the same vein, a population-based cohort study of nearly 220,000 Australian children found that a first dose of wP vaccine before 4 months old, rather than aP vaccine, was associated with 53% lower risk of hospitalizations for food-induced anaphylaxis between 5 and 15 years old (adjusted hazard ratio 0.47, 95% CI 0.26–0.83).[6] *In contrast with these findings, however, we found no evidence of a difference in admissions to hospital coded as asthma during the same study period (adjusted hazard ratio 1.02, 95% CI 0.94–1.12).[7]*  Previous studies have described differences in the immune effects arising from vaccination with wP versus aP vaccines in infancy. Compared to exclusive vaccination with aP which elicits a T-helper (Th)_2_-polarised immunophenotype, a first dose of wP induces relative Th_1_/Th_17_ immune polarisation, reducing the production of aP antigen-specific type 2 cytokines and down-regulating the synthesis of total IgE, and specific IgE responses against tetanus toxoid (TT), diphtheria toxoid (DT), pertussis toxin (PT), and food antigens.[7–10] *Moreover, enhanced PT-IgE and PT-IgG4 responses to filamentous haemagglutinin (FHA) and fimbriae 2/3 (FIM 2/3) after dTap boosting have been described in a subset of adults who had received primary infant vaccination with aP only doses, but not among those primed with a homologous wP schedule.[12]* |
| 6. Line 98 - The reviewer wonders what the rationale was for choosing 2 time points so close to each other. | We acknowledge that blood specimens at other time points would have been informative, but we needed to ensure parsimony of venepuncture on ethical grounds. The 7-month schedule point provides information about vaccine responses following completion of a primary schedule at 6 months. A sample immediately before the 6-month dose allows us to evaluate both the absolute antibody concentrations after 2 priming vaccine doses and also the relative (fold-)rise in concentrations following the 6-month vaccine doses |
| Results: 7.      Overall, the structure would benefit from more informative subheadings. | **Agree.** We have incorporated additional subheadings for the sections that describe the safety and parental acceptability findings. |
| 8. Line 229 - The supplementary document is overwhelming with too much information and little to no context besides a figure legend. Although I understand that these are important documentations, I wonder if all are really necessary for the readership of this journal and/or if another online repository of clinical trial data and a link wouldn't be more suitable. Also, Figure 2.1 is exactly the same as Figure 2 in the manuscript body. Figure 2.3 is interesting and should be mentioned since it could reflect the different kinetics of two different pertussis immunization schedules. It would also be very interesting to understand these dynamics at the 12-month or over time points, which would be a key point on durability of protection. | **Noted**   a. The differential kinetics of the novel mixed schedule versus the aP-only schedule will be reassessed pre and post DTaP boosting at ~18 months old. b. We added Figure 2.3 to the manuscript (labelled as Figure 3 in the revised submission). c. The supplement has been revised to remove tables and figures already reported in the manuscript, to have more consistent formatting, and to reduce the overall number of tables and figures. |
| 9. Line 239 - The authors should also comment on the lower seroconversion of Fim 2/3. | **Agree.** The following paragraph was added to the discussion (lines 385 to 387). “*IgG GMCs for FIM2/3 were low at 6 and 7 months in both groups. Infanrix-Hexa has no FIM2/3 antigenic content; in the absence of natural infection, a progressive decline in anti-FIM2/3 antibodies which are either maternally-derived or induced by a single dose of wP is expected.”* |
| 10. The statistical differences are difficult to interpret due to the complexity of the statistical methodology. I am curious if there is a simpler method available to summarize these differences. [Editors’ note: As above, the statistical analysis should follow the prespecified protocol/SAP.] | We are guided by the editor’s comment and report the study findings as stated in the SAP. |
| 11. Also, why was IgE responses to pertussis vaccine antigens not performed? | We previously observed a rise in anti-TT IgE in infants vaccinated with aP-only but not with mixed wP/aP (Holt et al, PMID: 27265452), so we aimed to replicate that finding. We note the scientific interest in anti-pertussis IgE responses. The clinical importance of IgE directed against pertussis antigens is unclear, and so this was not prioritised in the current protocol. All IgE responses were measured using the ImmunoCAP platform. To date, the authors are not aware of validated IgE assays for pertussis antigens using the ImmunoCAP assay. |
| Discussion:  12. Some study limitations are presented throughout the well-written discussion but could be all condensed in a unique paragraph. Can you also develop on how and what is going to be exactly evaluated in the assessment of T cell responses? | **We respectfully disagree.** The idea of using wP for allergy prevention is likely to be controversial (see reviewer 3). In the interests of scientific process, we believe it’s important to present the context and the limitations of the study thoroughly. We therefore propose to keep the limitations section relatively unchanged, apart from some additions suggested by reviewer 1.  With regards to future studies of T cell responses, we have now added to the discussion (lines 427 to 430): *Vaccine responses have been traditionally assessed by measuring antibody responses only. Pertussis-specific T-cell memory responses may be important in long-term protection induced by pertussis vaccines.[8] Future studies will compare the patterns of CD4+ T-cell polarisation in response to pertussis vaccine antigens in a subcohort of infants enrolled in stage one.* |

| **Reviewer #3 comments** [Numbering added by editor] | **Authors’ answers** |
| --- | --- |
| This manuscript reports the results of a clinical trial to assess the possibility that a single dose of a whole cell pertussis (wP) vaccine given before the acellular pertussis (aP) vaccine might prevent or reduce IgE responses to food allergens. However, the results revealed no benefit in terms of reduced IgE response to egg allergens and as expect the wP vaccine was associated with higher adverse events. | We thank the reviewer for their feedback and appreciate the time spent reviewing our manuscript. |
| Specific comments:   1. The wP vaccine was discontinued in most developed countries in the 1990s. While the aP vaccine is clearly inferior to the wP vaccine, especially at preventing nasal infection and transmission of B. pertussis, it seems a retrograde step to go back to wP, even for a single dose in the primary immunization schedule. Alternatives to the current aP vaccine, including attenuated vaccines, aP vaccines with novel adjuvants and less toxic wP vaccines are in development and these would appear to be a better solution to the less-than-ideal current aP vaccine. While the parents of the children in this trial may have indicated acceptance of the side effects associated with the wP vaccine, this is unlikely to the general view from health care professionals, regulators and parents globally. | **We note and welcome the reviewer’s opinion regarding the contemporary use of wP.**   a. We acknowledge the challenges of re-establishing wP in high income countries which have transitioned to aP, but we also note the major challenges of developing completely new pertussis vaccines. We have discussed these challenges elsewhere and now reference this (Snelling & Waddington, PMID: 25426699). We note that from a global perspective, wP remains WHO’s preferred pertussis vaccination strategy, is readily available and licensed, and remains the most commonly used pertussis vaccine strategy by far. b. The original submission acknowledged the remaining uncertainty about the observed parental acceptance of the mixed strategy, and whether this can be generalised to the broader population of parents. The following sentence is now added to the discussion section (lines 478 to 481): *We note that many parents were motivated to participate because of a desire to prevent food allergy in their infants. Further evidence is required to understand population-level acceptability of this approach beyond the study population.* |
| 2. Previous studies have reported that aP vaccines do not boost IgE response to common allergens, despite promoting Th2 and IgE response to aP vaccine antigens. The current report references previous studies that have suggested that Australian children who received wP, rather than aP, vaccine as their first immunization had less IgE-mediated food allergy. So the question remains, did immunization with aP vaccine only promote IgE response to allergens (over that which might be developed irrespective of vaccination) or did the wP vaccine induce some "protective" effect against general food allergen-induced IgE and if so, how. If the wP vaccine is "protective", this boils down to giving children a reactogenic vaccine to reduce the incidence of food allergy. | **We agree.** If a first dose of wP is found to afford protection against certain IgE-mediated food allergies, the immune mechanisms should be further elucidated. To this end, a subcohort of infants enrolled in stage one has been consented for future mechanistic (transcriptomic and immunological) studies. |
| 3. The underline hypothesis for this study seems to have been based on the premise that the Th2 and associated IgE response induced by the aP vaccine would be skewed more to Th1/Th17 with a primary dose of the wP vaccine and this would reduce/eliminate the IgE responses. However, the study has not assessed or reported any data on T cell responses from the clinical trial. This is a significant omission and weakens the impact of the study. | **Noted.** T-cell responses will be evaluated in a subcohort of infants enrolled in stage one. |
| 4. The presentation of the results, especially the narrative in the text of the results section is weak, with very limited commentary on the data, their meaning or significance. | **We respectfully disagree.** The authors consider that the results section should present the data and the analyses, and should not be contaminated with our subjective interpretation of the data, their meaning, or significance. This was reserved instead for the discussion section, which we note has been praised by reviewers 2 and 4. |
| 5. The authors use the term "seropositive/seroprotective threshold" when referring to IgG responses to B. pertussis antigens. There is no correlate of protective immunity against B. pertussis, so could they please explain what is a seroprotective concentration of antibody and against what antigen(s)? | **We agree.** As presented in the methods section, seroprotective thresholds are defined for DT and TT, but not for pertussis antigens.   a. **The original methods section reads**: “The threshold for seropositivity was defined as concentrations ≥ 5 IU/mL (5000 mIU/mL) for pertussis antigens (i.e., PT, filamentous haemagglutinin [FHA], pertactin [PRN], and fimbriae type 2 and 3 [FIM2/3]).[15] Long-term seroprotective concentrations for TT and DT were defined at ≥ 100 mIU/mL.[16,17]” **No changes are made.** b. The following edits were made to the results section (lines 277 to 279): *At 7 months old, 68 /69 infants in the aP-only group (99%) and 66/67 infants in the mixed schedule group (99%) had PT-, FHA-, and PRN-IgG concentrations above the specified seropositivity threshold, as well as TT- and DT-IgG above the specified seroprotective threshold.* c. The following edits were made to the discussion section (378 to 382): *While at 7 months old almost all (99%) serum responses to DT and TT met their specified seroprotective thresholds and the IgG responses to FHA and PRN met the specified seropositivity cut-off in the two treatment groups, PT and FHA-IgG concentrations immediately before the 6-month aP dose were lower in the mixed schedule group compared to the aP-only group.* |
| 6. The study has used a wP vaccine from PT Biofarma, Indonesia (and this is not mentioned until the discussion section). Detailed information and/or references to studies using this vaccine should be provided in the Methods section of the manuscript. | **We disagree.**   a. The methods section (lines 127 to 130 of the original submission) provides information on wP and aP vaccine manufacturers: “Infants were assigned in a 1:1 ratio to receive either the intervention (pentavalent wP combination vaccine: (DT, TT, wP, Haemophilus influenzae type b [Hib], and hepatitis B [HepB] vaccine; DTwP-Hib-HepB, **Pentabio, PT Bio Farma, Indonesia**) or the comparator (hexavalent aP combination vaccine, which includes inactivated poliovirus vaccine [IPV] types 1, 2, and 3 in its formulation: DTaP-Hib-HepB- IPV, Infanrix Hexa, GlaxoSmithKline, Australia) at approximately 6 weeks old. b. The current version of the manuscript cites a prospective, randomised, double blind, multicenter, phase III study of Bio Farma DTP-HB-Hib vaccine conducted in Jakarta and Bandung (Indonesia) by Rusmil et al (Ref 15). |

| Reviewer #4: Alex McConnachie, Statistical Review [Numbering added by editor] |  |
| --- | --- |
| The paper by Pérez Chacón et al reports on the initial phase of the larger OPTIMUM trial, in which the immune responses and safety of two vaccination schedules are compared. This review considers the statistical aspects of the paper. Overall, I found the paper to be nicely written, and the statistical methods and application seem very good. The data presentation is quite clear, with some very nice graphs. I do have a few comments, which are fairly minor. I shall describe these in the order in which I encountered them in the paper. | We thank Prof McConnachie for their feedback and appreciate the time spent reviewing our manuscript. |
| 1.      The primary analysis is by intention to treat. For a non-inferiority trial, some would argue that the primary analysis should be for a per-protocol population, though I think it is fine as long as both are reported. The PP results are somewhat hidden away in the supplements, and do not appear to be commented on in the paper. I think a few words as to whether the results are consistent, and a signpost to the results, would be worth adding. | **We agree.** While PP is criticised for failing to conserve the randomisation, it can be important adjunct to assessing non-inferiority where there are frequent post-randomisation events/ missing data. It was pre-specified and reported for completeness. The following sentence is added to the results section (e.g., Immunogenicity and IgE-mediated immune responses subsections): *Despite exclusions, the results were consistent in the PP analysis set (lines 289 and 290; 309 and 310).* We have also cross-referenced the pages where the PP analyses can be found in the supplement. |
| 2.      The methods section of the paper describes SAEs by the usual definition, including congenital anomalies and birth defects. Whilst technically not wrong, does this part of the definition really apply in this study? | **Noted.** We nonetheless abide by the Australian National Health and Medical Research Council standard SAE definition. |
| 3.      There is nothing I could see about the choice of sample size in the paper, and looking at the protocol in the appendix, there is also nothing to justify the choice of 150 for this first phase of the study. There is a link and reference to the SAP at the start of the methods section, but again, nothing immediately obvious about this. How was this decided upon? | **Agree.** We now add the following paragraph to the statistical analysis section (lines 217 to 223). *Based on previous clinical/immunological data, we determined that a sample size of 150 infants, allocated 1:1 to each schedule, would provide a power of at least 80% to detect a 20-percentage point decrease in the proportion of participants with detectable TT-specific-IgE at 7-months old between infants primed with mixed wP/aP and aP-only. This controls the significance level (probability of falsely declaring a difference if none existed) at 5% for a one-sided hypothesis test (wP/aP less than aP-only) and assumes that the proportion of participants with detectable TT-IgE specific-IgE at 7-months old is at least 25% in the aP-only arm.* |
| 4.      The statistical methods used seem good. As a non-Bayesian, there was quite enough in the statistical methods for my taste, but other readers may wish to see more detail. The protocol seems to focus more on the second phase of the study as a whole, and it is only in the SAP that I could find more details. Should this be included in the supplements, and referenced more explicitly in the text of this paper? One thing that I thought was missing from the statistical methods was some information about the choice of prior distributions. I did find this on digging into the SAP, but a quick mention, with reference to the SAP, could be made in the main text. | **Noted.** The statistical analysis section was expanded in section 1 of the supplement. |
| 5.      In non-inferiority trials, a justification for the non-inferiority margin is required, and this is provided by reference 18 in the paper. However, when trying to check this, the link did not work for me. (I did get to the document by a different route, and can confirm that the margin chosen is appropriate). | **Noted.** On March 23^rd^, 2024, we tested the link (<https://cdn.who.int/media/docs/default-source/prequal/vaccines/who-trs-1004-web-annex-9.pdf>) on different browsers and had no issues retrieving the document. Since this might not be the case for all the readers, we are providing a different route to access the same document (<https://www.who.int/publications/m/item/WHO-TRS-1004-web-annex-9>). |
| 6.      Reading the paper, it is not until you reach the end of the discussion that plans for the second phase become clear. It might help, for the sake of context, to describe this earlier on. Note, the paper states a sample size of up to 2000 for the second stage, but the protocol and SAP talk about a maximum of 3000. Is the paper in error? | **Agree.** This is an error. The methods section is now edited as follows (lines 136 to 139): Stage two has been designed to assess the primary endpoint of IgE-mediated food allergy, with less intensive follow-up, but with sufficient sample size (up to **3000** infants, including the first 150 enrolees in stage one) to provide appropriate levels of statistical power. |
