# Supplementary material for: Immunogenicity, reactogenicity, and IgE-mediated immune responses of a mixed whole-cell and acellular pertussis vaccine schedule in Australian infants: A randomised, double-blind, noninferiority trial
Source: PLoS Med. 2024 Jun 10;21(6):e1004414. doi: 10.1371/journal.pmed.1004414 (PMC11198910; doi:10.1371/journal.pmed.1004414)
Supplement: Attachment — Submitted filename: 13-04_6m_plos_med_revisions_v3.0.docx [file pmed.1004414.s004.docx]

Editorial requests:

1. Abstract: please state the primary outcome(s) clearly and specify the analysis population (eg, ITT).

***Re: done (lines 50 and 60; clean version).***

*Primary outcome: Ahead of ascertainment of the primary clinical outcome of allergist-confirmed IgE-mediated food allergy by 12 months old […]*

*Analysis population: The primary analyses were based on intent-to-treat (ITT); secondary per-protocol (PP) analyses were also performed.*

2. Abstract: please include data on the most common AEs and report the 7 SEAs within the findings section of the abstract, per CONSORT for Harms (<https://www.equator-network.org/reporting-guidelines/consort-harms/>).

***Re: done (lines 68 to 70; and lines 71 to 73, clean version).***

*Most common AE: At 6 weeks old, irritability was the most common systemic solicited reaction reported in wP (65 [88%] of 74) versus aP (59 [82%] of 72) vaccinees.*

*Report the 7 SAEs within the finding section of the abstract: There were 7 SAEs among 5 participants within the first 6 months of follow-up; on blinded assessment, none were deemed to be related to the study vaccines.*

3. Abstract: please be sure that the past tense is consistently used, to reflect the fact that phase 1 of the trial is complete (this pertains to the Author summary [‘What did the researchers do and find’ section] as well).

***Re: done (abstract and author summary: why did the researchers do and find?)***

*Methods (abstract; lines43 to 49): OPTIMUM is a Bayesian, two-stage, double-blind, randomised trial. In stage one, infants* ***were assigned*** *(1:1) to either a first dose of a pentavalent wP combination vaccine (DTwP-Hib-HepB, Pentabio PT Bio Farma, Indonesia) or a hexavalent aP vaccine (DTaP-Hib-HepB-IPV, Infanrix hexa, GlaxoSmithKline, Australia) at approximately 6 weeks old. Subsequently,* ***all infants received*** *the hexavalent aP vaccine at 4 and 6 months old as well as an aP vaccine at 18 months old (DTaP-IPV, Infanrix-IPV, GlaxoSmithKline, Australia). Stage two is ongoing and follows the above randomisation strategy and vaccination schedule.*

*What did the researchers do and find (lines 86 to 93; clean version):*

- ***In the first phase*** *of a two-stage, double-blind, non-inferiority trial, Australian-born infants* ***were randomised*** *to receive a first dose of wP versus aP at approximately 6 weeks old; in both study groups aP vaccine is given at 4 and 6 months old.*
- ***Stage (phase) one examined*** *the immunogenicity, reactogenicity, and IgE-mediated immune responses of the mixed vaccine schedule (wP/aP/aP) versus the standard aP-only vaccination strategy (aP/aP/aP) in a cohort of 150 infants; stage two is ongoing and was designed to ascertain the development of food allergy by 12 months old (primary outcome) in a cohort of up to 3000 infants.*

4. Abstract (minor): please remove the comma in line 63.

***Re: done.***

5. Data availability statement: Please remove this paragraph from the final text file (it will be included in the metadata that precedes the published paper).

***Re: done.***

6. Methods: lines 170-171: Does this sentence refer to paracetamol (“Two additional doses 6-hour apart were recommended, but not observed by the researchers.”)? Please clarify.
   ***Re: done.***

***Two additional doses of paracetamol*** *6-hour apart were recommended, but not observed by the researchers (lines 178 and 179; clean version).*

7. Results: please present the data as n (%) of N throughout. For example, at line 273, “72 (96%) of 75 infants in the aP-only schedule group…”

***Re: done.***

8. Discussion: line 464, please add ‘To our knowledge’ (this should be done wherever there is a claim about primacy).

***Re: done (lines 485 to 487; and 489 to 490; clean version)***

*However, to our knowledge, except for analyses carried out in Australia,[26] Belgium,[9] and the US,[42] TT-specific IgE responses have been measured using RAST or other serological methods.*

*To our knowledge, this study represents the first prospective randomised comparison of a novel mixed wP/aP versus aP-only schedule.*

9. Figures 2 and 3: please remove ‘boxplots’ from the figure title. Please also add the definitions of ‘aP’ and ‘wP’ to the figure legend.

***Re: done (lines 276 to 279; clean version).***

10. Figures 5-7: please indicate in the figure legends what the number above each bar indicate (n/N).

***Re: done (lines 343 to 365; clean version) – Please see our answer to the eleventh editorial request.***

11. Figures 5 and 6: Please remove the “6 month” heading from the top of the figures, as this is indicated in the figure titles (and is not included in Fig 7).

***Re: This editorial request has been handled by editing Fig 5, 6, and 7 titles (see below). No further changes to the figures have been made.***

**Fig 5. Maximum injection site reaction size in the 7 days after the 6-week, 4-month, and 6-month vaccine doses (lines 343 and 344).** *aP, acellular pertussis vaccine; wP, whole-cell pertussis vaccine; 13vPCV: 13-valent pneumococcal conjugate vaccine; DTaP-Hib-HepB-IPV, hexavalent aP vaccine (diphtheria, tetanus, aP, Haemophilus influenzae type b, hepatitis B, and inactivated poliovirus types 1, 2, and 3 vaccine). Above each bar (n/N) we indicate the number of infants reporting any injection site reaction > 0 mm (numerator; n) versus the total number of infants reporting on injection site reactions ≥ 0 mm (denominator; N).*

**Fig 6. Injection site pain in the 7 days after the 6-week, 4-month, and 6-month vaccine doses (line 350).**

*aP, acellular pertussis vaccine; wP, whole-cell pertussis vaccine; 13vPCV: 13-valent pneumococcal conjugate vaccine; DTaP-Hib-HepB-IPV, hexavalent aP vaccine (diphtheria, tetanus, aP, Haemophilus influenzae type b, hepatitis B, and inactivated poliovirus types 1, 2, and 3 vaccine); pain intensity, mild (minor reaction on touch or does not interfere with daily activities); moderate (cries / protests on touch or interferes with daily activities); severe (cries when limb is moved/spontaneously painful or prevents daily activities). Above each bar (n/N) we indicate the number of infants reporting any pain at the injection site (i.e., mild, moderate, or severe pain; numerator; n) versus the total number of infants reporting on pain at the injection site (none, mild, moderate, or severe pain; denominator; N).*

**Fig 7. Highest intensity grade for systemic reactions after the 6-week, 4-month, and 6-month vaccine doses (lines 359 and 360).**

*aP, acellular pertussis vaccine; wP, whole-cell pertussis vaccine; grade 1: easily tolerated; grade 2, interferes with normal everyday activities; grade 3, prevents normal everyday activities or requires significant medical intervention. Above each bar (n/N) we indicate the number of infants reporting systemic reactions grade 1 and above (numerator; n) versus the total number of infants reporting on systemic reactions (none, grade 1, grade 2, or grade 3; denominator; N).*

12. Table 1: please define TAFE in the footnote.

***Re: done (line 264).*** *TAFE: technical and further education.*

13. References: for relevant refs (URLs) change ‘cited’ to ‘accessed’ and move the accessed date to after the URL; add date accessed for refs 24, 25, 27, 28
    ***Re: done.***
14. As a reminder, we ask every co-author listed on the manuscript to fill in a contributing author statement. If any of the co-authors have not filled in the statement, we will remind them to do so when the paper is revised. If all statements are not completed in a timely fashion this could hold up the re-review process. Should there be a problem getting one of your co-authors to fill in a statement we will be in contact. Please do not add or remove authors without first agreeing this with the handling editor.

***Re: No additional edits are applicable.***

Additional edits

1. Acknowledgement section (lines 663 to 677; clean version): This section has been edited to include a formal scholarly acknowledgment of the traditional custodians of the land and waters of (Western) Australia.

*We acknowledge Aboriginal and Torres Strait Islander People as the Traditional Custodians of the land and waters of Australia. We also acknowledge the Nyoongar Wadjuk, Yawuru, Kariyarra and Kaurna Elders, their people and their land upon which the Telethon Kids Institute is located, and where stage one of the OPTIMUM trial was conducted. We seek their wisdom in our work to improve the health and development of all children.*
